# Computational Insights into the Adsorption of Ligands on Gold Nanosurfaces

**DOI:** 10.1021/acs.jpca.3c05560

**Published:** 2023-11-22

**Authors:** Sveva Sodomaco, Sara Gómez, Tommaso Giovannini, Chiara Cappelli

**Affiliations:** Scuola Normale Superiore, Classe di Scienze, Piazza dei Cavalieri 7, 56126 Pisa, Italy

## Abstract

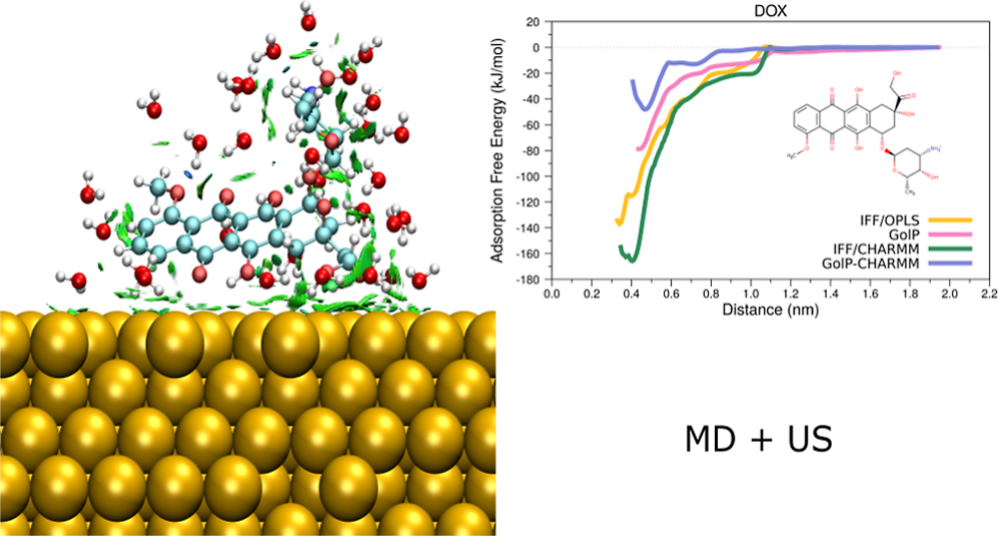

We study the adsorption process of model peptides, nucleobases, and selected standard ligands on gold through the development of a computational protocol based on fully atomistic classical molecular dynamics (MD) simulations combined with umbrella sampling techniques. The specific features of the interface components, namely, the molecule, the metallic substrate, and the solvent, are taken into account through different combinations of force fields (FFs), which are found to strongly affect the results, especially changing absolute and relative adsorption free energies and trends. Overall, noncovalent interactions drive the process along the adsorption pathways. Our findings also show that a suitable choice of the FF combinations can shed light on the affinity, position, orientation, and dynamic fluctuations of the target molecule with respect to the surface. The proposed protocol may help the understanding of the adsorption process at the microscopic level and may drive the in-silico design of biosensors for detection purposes.

## 1 Introduction

The understanding, at the molecular level, of the interaction between ligands and nanostructured surfaces is very important in the current research, especially because it can drive the design of novel sensing devices for the detection and identification of chemical and biological agents, ranging from small drugs to viruses and bacteria.^1^ In many different multidisciplinary areas of nanoscience and nanotechnology,^2,3^ systems composed of a target molecule, an inorganic substrate, and an aqueous solvent are gaining increasing attention due to their potential applications. For instance, noble metals and graphene-based engineered materials provide a powerful tool to combine tunable spectral selectivity and enhanced sensitivity, which can be exploited in the growing fields of biosensing.^4−9^ Nevertheless, the design and optimization of nanotechnological applications benefit from understanding the molecular factors that drive the recognition and binding of the molecule on the nanosurface. Notably, the investigation of the structural, dynamic, and interaction features of molecule-inorganic surface-water systems is challenging both experimental and theoretical research fields.

The intrinsic complexity of molecule–surface assemblies, which span a wide range of time and length scales, can only be partially investigated by means of experimental measurements. In fact, in the protein case, experiments aimed at probing the interface between the protein and the surface in an aqueous environment are extremely demanding and provide indirect information at nanometer resolution, leading to uncertainty on the location and orientation of the adsorbed biomolecules and on the role played by polyelectrolytes and surfactants at the aqueous interface.^10,11^ In addition, experimental techniques like surface plasmon resonance spectroscopy and quartz crystal microbalance can provide adsorption free energies of peptides on gold surfaces,^12,13^ but they are still limited to single molecules. Similarly, other techniques like single-molecule force microscopy (SMFM) and isothermal titration calorimetry afford valuable information that can be used to indirectly estimate adsorption free energies (AFE) based on desorption forces and thermodynamic measurements and provide a preliminary comparison with direct experimental and computational data.^14^

Due to the lack of (direct) experimental data for the majority of molecular species, computational techniques can effectively complement experimental studies to atomistically investigate molecule/surface/water interfaces and evaluate the binding affinity to nanosurfaces.^11,12,15,16^ A great effort in this direction is already underway, and computational chemistry offers new insights into recognition and binding processes and explains how molecules, especially biomolecules, interact with different kinds of surfaces.^17,18^ However, as pointed out by Martin et al.,^19^ “there are no proven models for describing biointerfaces” because of the lack of experimental information to validate results, and this translates into always newly developed methodologies for the simulation of molecule–surface interactions trying to better describe and predict adsorption phenomena.

Simulating water-solvated molecules in the presence of inorganic surfaces requires an accurate description of each component of the system and a robust sampling method.^20,21^ Extensive sampling methodologies are currently being developed and applied to explore the complex phase space of (bio)molecules/surfaces. Classical molecular dynamics (MD) simulations are by far the most used and suitable method to reveal the interesting dynamical features of this kind of aqueous system and to gain insight into adsorption events on a molecular scale. In this regard, consistent force fields (FFs) have been built to describe the properties of adsorbates and plasmonic surfaces in water, both separately and as a whole.^20,21^ Until now, there are few computational protocols^16^ able to model and simulate molecules of different natures and sizes interacting with various inorganic surfaces in solution.

In this work, we propose a computational strategy to study, at an atomistic level, the adsorption of small- and medium-sized molecules on nanostructured plasmonic surfaces in an aqueous environment. To that end, we used MD trajectories of the adsorption process of several adsorbates on gold and estimated the AFEs. We focus on the parameters affecting the molecule–surface interaction, i.e., the average distance of the molecule from the surface, its orientation once it is adsorbed on it, and the nature of the interaction, as mediated by the surrounding water molecules. Such a computational protocol may help the understanding of the adsorption process at the microscopic level and the in silico design of (bio)sensors for detection purposes.^22−24^

## 2 Methods

The simulation of interfacial systems requires the application of a computational strategy based on three main aspects. First, a realistic representation of the structure of the molecule, the surface, and the solvent can be achieved by adopting fully atomistic approaches. Second, the necessity of using classical approaches for the description of the physicochemical properties of these systems.^21^ Indeed, dealing with their complexity, which covers a broad range of time and length/size scales, going from femtoseconds to milliseconds and from nanometers to micrometers, prevents the use of quantum mechanical methods. Within this framework, a suitable set of parameters is needed to properly reproduce the atomic properties of each component and its interaction with the others. Third, the exploration of the conformational phase space of the molecule and the surrounding solvent upon the surface by means of a robust sampling method, for instance, MD simulations. The thermodynamic description of adsorption processes thus benefits from the combination of accurately parametrized FFs and well-sampled configurations, which account for enthalpic and entropic contributions to adsorption.

Potential of mean force (PMF) methods like umbrella sampling (US)^25^ can be applied to such systems to recover information for the travel of the molecule from the bulk solution to the surface and to obtain adsorption free energies. US is used as a benchmark reference for evaluating the performance of other free-energy computational protocols^26−28^ and has also been employed in the study of adsorption processes.^29^ Despite its practical application, the usage of the US is often limited by its elevated computational cost since various overlapping windows, sufficiently equilibrated, must be prepared.

In this section, the next steps are addressed: a short description of the chosen FFs to treat each part of the system, the building of the system in relation to its characteristic features, and the computational procedure to be followed in MD and US simulations.

### 2.1 Force Fields for the Gold Surface

In the literature, different FFs are available to describe gold surfaces, and most of them rely on classical Lennard-Jones (LJ) potentials to include nonelectrostatic interactions.

Among the first optimized sets of LJ parameters developed to model mixed interfaces, there is the parametrization proposed by Heinz et al., incorporated in the INTERFACE FF (IFF) for inorganic nanostructures.^30,31^ Within IFF, the face-centered cubic (fcc) metal consists of charge-neutral atoms with repulsive and dispersive van der Waals interactions. Therefore, they can rearrange their positions, thus allowing the modeling of different crystallographic facets and shape constructs. The model comprises just one atom type for the metal atoms; that is, only one set of LJ parameters for each metal. IFF parameters are the result of a parameter validation process only involving properties of pure metal and nonbonded interactions. Therefore, the interaction of the surface with other components of the interface is considered to be mainly dictated by the metal itself. Such parameters are transferable since the functional form of the energy is compatible with a broad range of “organic” FFs (AMBER,^32^ CHARMM,^33^ and OPLS/AA^34^ among them), thus implying that standard combination rules are sufficient to deal with interfaces of various kinds.^20,35^ In its original formulation, IFF lacks the inclusion of explicit metal polarization effects, since the parametrization against experimental data should, in principle, make them implicitly accounted for. Nevertheless, Heinz et al. have recently introduced a core–electron polarizable LJ potential to describe gold surfaces, whose effectiveness has been proven in the specific case of charged ions and peptides.^36^

GolP FF,^37^ related to the previously established OPLS/AA FF, and GolP-CHARMM,^38^ developed on the basis of the widely used CHARMM family of FFs, economically incorporate polarization effects using the rigid-rod model proposed and implemented by Iori and Corni^39^ in 2008. GolP was specifically developed for Au(111) surfaces, whereas GolP-CHARMM was extended to treat also Au(100).^40^ Unlike IFF, GolP and GolP-CHARMM do not include intrasurface Au–Au interaction terms. As a result, the gold surface is kept frozen, which disables any potential gold lattice deformation and prevents the evaluation of the surface energies or intrinsic properties of the metal. An important aspect of GolP FFs is the introduction, for each gold atom, of two LJ virtual sites occupying the hollow sites of the gold surface that favor on-top adsorption, as suggested by DFT calculations.^37^ With respect to the IFF, GolP FFs contain specific parameters to describe the interactions between the adsorbate and gold. This aspect prevents transferability to other bio-oriented FFs.

GolP and GolP-CHARMM FFs have been specifically parametrized so as to give a balanced description of the gold-molecule versus gold–water interactions, which is essential since the adsorption of a molecule on a surface is actually a competition between the molecule and the water molecules for that surface. Then, special attention has been paid to the modeling of water molecules adsorbed on gold, given that water structuring and layering above both Au(111) and Au(100) surfaces can strongly affect the adsorption of biomolecules.^41^

In this work, nonpolarizable and polarizable IFF, as well as GolP and GolP-CHARMM, have been tested to study the adsorption processes on Au(111).

### 2.2 System Preparation and Simulation Setup

**Figure 1:**
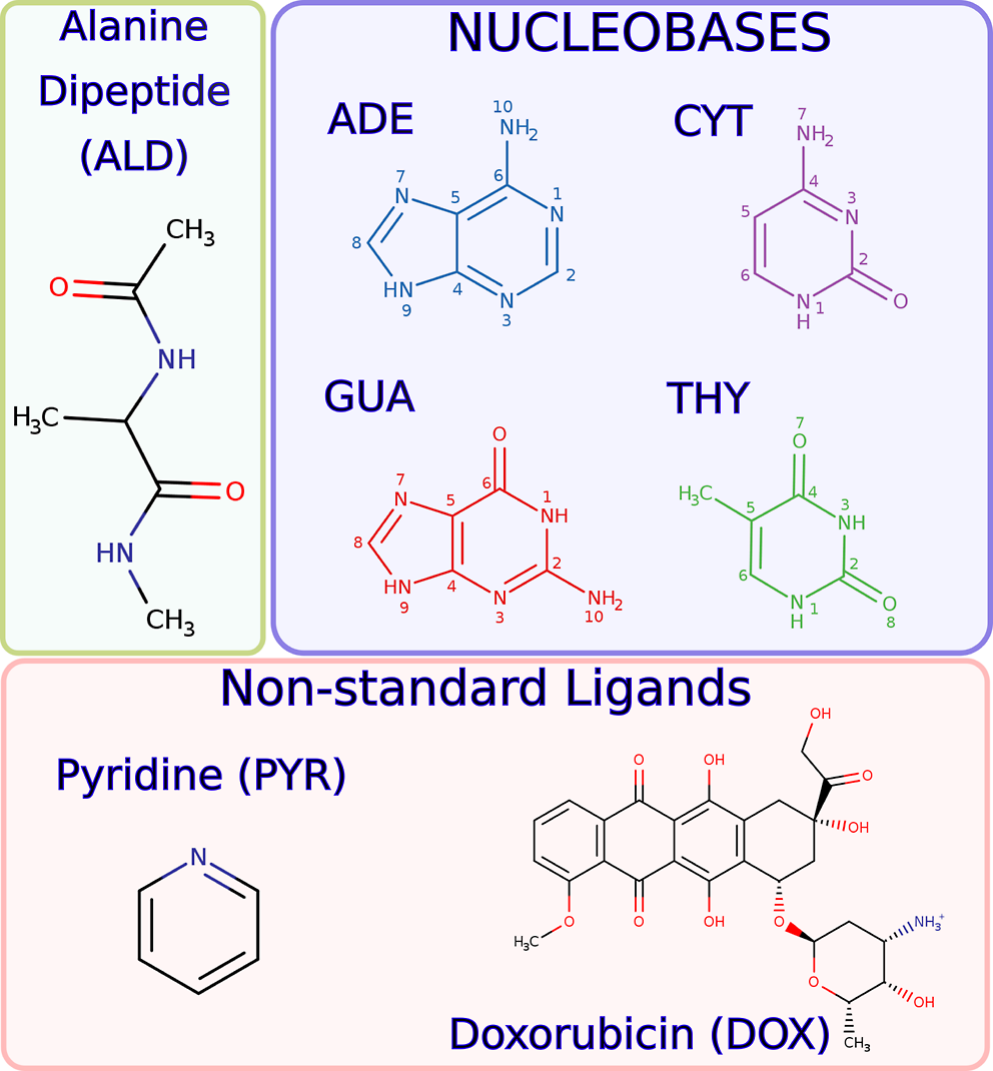
Structures and atom numberings of the adsorbates studied in this work.

We have investigated the adsorption on gold nanostructures of the seven molecules shown in Figure 1: alanine dipeptide (ALD) as a representative of peptide structures, the four DNA nucleobases [adenine (ADE), guanine (GUA), cytosine (CYT), and thymine (THY)], and two nonstandard ligands [pyridine (PYR) and doxorubicin (DOX)].

The gold nanostructure is here modeled as a planar surface under the assumption that neglecting the curvature of the nanoparticle/nanoaggregate represents a good approximation of the experimental setup. Among the various crystal surfaces, we have selected Au(111) facets, which are the most stable and therefore the most abundant in nanoparticles.^42^ The gold slabs are generated using the atomic simulation environment (ASE) Python module,^43^ which sets up the lattice constant, the crystal packing (fcc111 in this case), the required dimensions, and hence the number of layers.

Optimized structures (at the DFT level, B3LYP/DZP) of the seven molecules are taken as the starting point to obtain parameters and charges compatible with the FFs employed to describe the gold surface. To this aim, LigParGen^44^ and CGenFF^45,46^ web servers are used, respectively, to generate OPLS/AA parameters to be joined with IFF and GolP FFs and CHARMM parameters to be used with IFF and GolP-CHARMM. This procedure allows us to test the performance of different combinations of existing force fields for the molecule and the gold surface and avoid expensive and time-consuming manual reparameterization. The standard nonpolarizable SPC^47^ and the TIP3P^48^ water models for the solvent are employed with OPLS/AA and CHARMM FFs, respectively.

### 2.3 Molecular Dynamics

**Figure 2:**
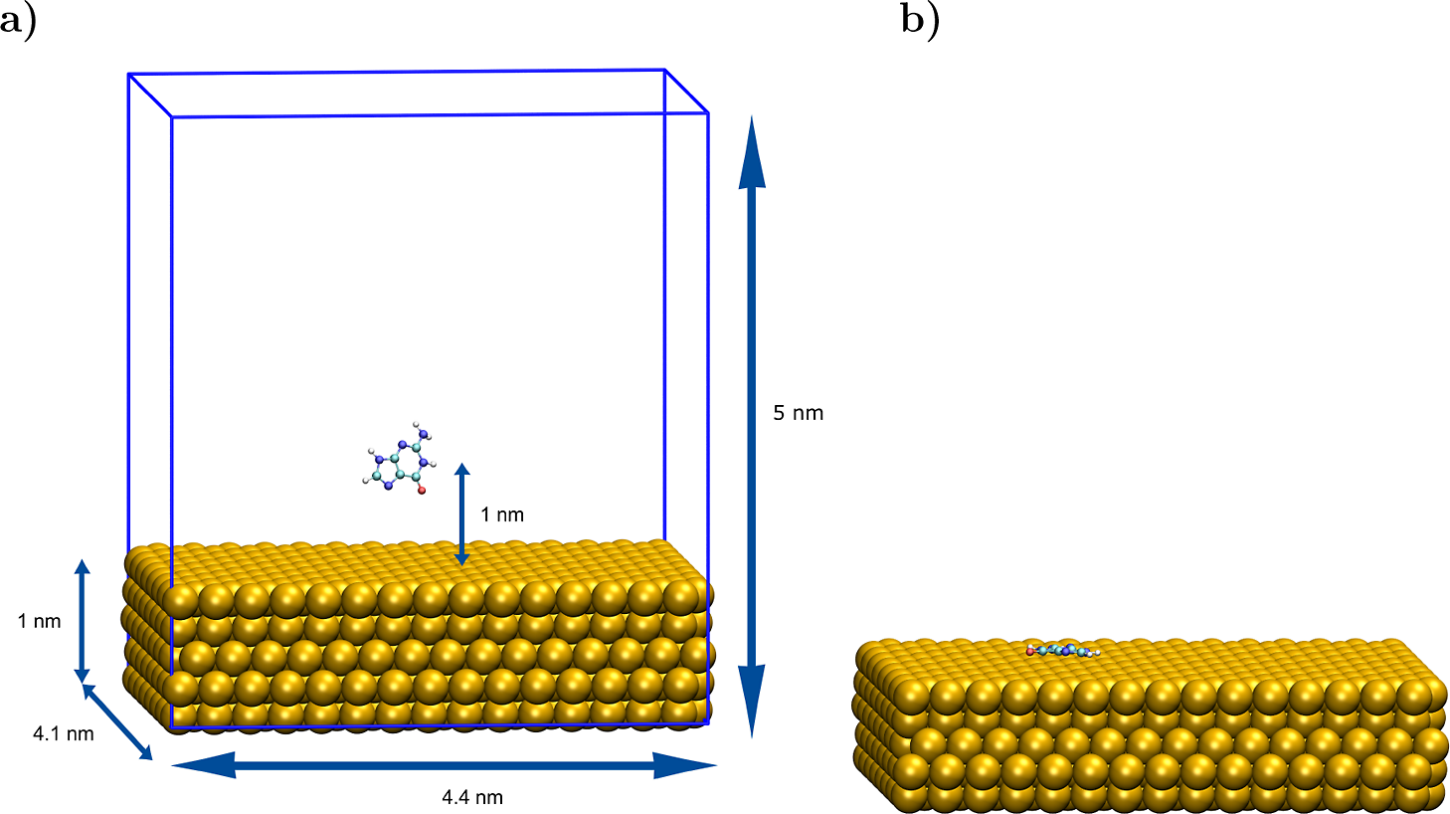
(a) Initial and (b) final configurations of guanine on gold. The system is enclosed in a 3D simulation box (blue lines). Water molecules and ions have been omitted for clarity. In the favorable adsorption geometries, all molecules studied here (see Figure 1) are adsorbed parallel (slightly tilted) to the surface.

MD simulations are performed in the GROMACS 2020.4 software.^49^ A 4.4 × 4.1 × 1.0 nm metal slab (5 layers thick so as to mimic the structure of a metal nanoparticle) is enclosed in a simulation box of 5 nm (7 nm in the DOX case) height. The metal slab comprises 1200 real lattice atoms that correspond to simulating 1200 particles within the nonpolarizable IFF, 2400 within its polarizable version, and 3360 in the GolP and GolP-CHARMM cases. The studied molecule is placed in the center of a 3D simulation box at 1 nm (1.5 nm for doxorubicin) from the top of the surface in a random initial conformation (see Figure 2a). The molecule/surface system is solvated with more than 2000 water molecules, and sodium and chloride ions are included in the box to neutralize the system and attain a physiological 0.154 M concentration. The solvated system is then subjected to steepest-descent energy minimization in order to remove clashes between the atoms and ensure starting from a reasonable structure in terms of geometry and solvent orientation. After the energy was minimized, a 2 ns simulation was performed to adjust the water density in the *NPT* ensemble at a pressure of 1 bar. Afterward, a 15 ns MD production is conducted in the *NVT* ensemble (time step of 2 fs) at 300 K using a Nosé–Hoover thermostat^50^ with a coupling constant of 2 ps. During these steps, the bond lengths of hydrogen atoms are constrained using the LINCS^51^ algorithm. Particle Mesh Ewald^52^ electrostatic summation is cutoff at 10 Å, and a force-switched cutoff starting at 9 Å and ending at 10 Å is used for LJ nonbonded interactions. Periodic boundary conditions are adopted in all simulations.

### 2.4 Umbrella Sampling

For the purpose of estimating the AFEs, the 1D US technique is applied during the adsorption/desorption processes of each adsorbate. Notice that, for some systems, like molecules embedded in lipid bilayers, it is important to consider the conformational freedom of the solute to guarantee proper sampling. As exposed by Jambeck and Lyubartsev^53^ and also in earlier works,^54−56^ this can be done by exploring more than one collective variable/coordinate, which is, indeed, the philosophy of 2D US calculations. Notwithstanding, such techniques are more computationally demanding than the standard 1D US.

In this work, starting configurations of each system for 1D US are sampled through steered MD simulations, in which the molecule is pulled away along the *z*-direction, i.e., perpendicular to the gold surface, from the previously obtained adsorbed state to the free state in solution with a rate of 0.5 nm per ns. 30–50 configurations are extracted along the trajectory and equilibrated at 300 K. Finally, each window is simulated for 5 ns in the *NVT* ensemble, restraining the center of mass (COM) of the molecule in the *z*-direction with a harmonic constant of *k* = 2000 kJ mol^–1^ nm^–2^, but allowing it to explore the *xy*-plane. Additional configurations and/or differently restrained windows are added whenever needed to improve the sampling. The weighted histogram analysis method (WHAM),^57^ implemented in GROMACS, is used as a postprocessing method to obtain the adsorption Helmholtz free energy profile, taking the free molecule in solution as the reference state and using a bootstrapping method (*N* = 100) to estimate error bars. 2D US test calculations for the adsorption of DOX indicated that even if the sampling is done with further degrees of freedom, the conformation of the molecule in the adsorption minima remains unaltered with the anthraquinone rings parallel to the surface while the rest of the molecule freely moves.

To further investigate the possible molecular basis of the adsorption mechanism, key points are extracted from the adsorption profiles, and non-covalent interaction (NCI)^58^ plots are constructed from the promolecular densities using the NCIPLOT4 program.^59^

## 3 Results

**Figure 3:**
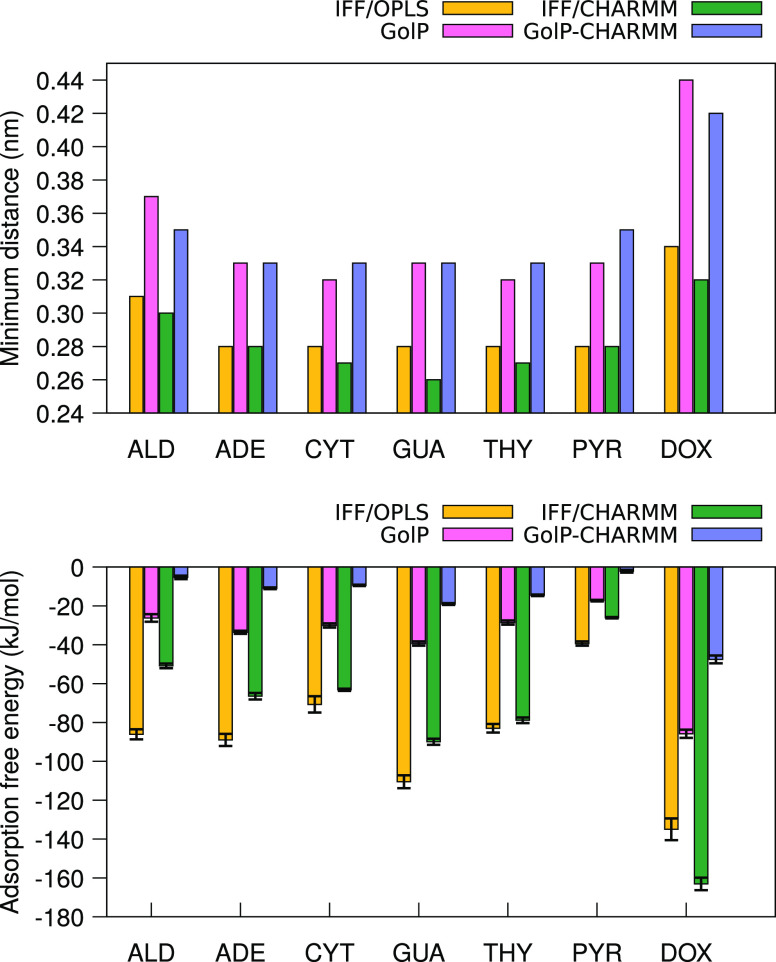
Performance of each combination of force fields for the seven studied molecules in estimating minimum distances from the gold surface, *d*_min_ (top clustered histogram), and adsorption free energies, Δ*E*_ads_ (bottom clustered histogram), with the corresponding error bars.

In this section, we report the results obtained with the methods explained in Section 2. Briefly summarized, the adsorption process of the set of molecules in Figure 1 on a gold surface was studied through 15 ns MD simulations. Example structures for GUA in its initial and final configurations are presented in Figure 2. Starting from the “adsorbed” configuration, the binding energies were calculated by using the US technique in a path normal to the metal surface, mimicking the deattachment procedure until the molecule was free again. The obtained adsorption energies, Δ*E*_ads_, and the minimum distance values, *d*_min_, are collected in Tables 1 and S1, and for the sake of comparison, they are plotted in Figure 3. Overall, all molecules present an affinity to the surface (Δ*E*_ads_ < 0), and each one lies horizontally (at around 0.30–0.40 nm) with respect to the surface, revealing some kind of strong interactions with the metal that are further studied by means of NCI analyses. In what follows, we will comment on the findings for every particular subset: alanine dipeptide, nucleobases, and nonstandard ligands.

**Table 1:** Computed Average Adsorption Free Energies, Δ*E*_ads_, and Minimum Distances, *d*_min_, for Adsorbed Molecule/Au(111) Systems of Figure 1, Using OPLS/AA for Each Molecule and IFF and GolP for the Gold Surface^a^

Ligand	IFF		GolP		other works
	Δ*E*_ads_	*d*_min_	Δ*E*_ads_	*d*_min_	
	(kJ/mol)	(nm)	(kJ/mol)	(nm)	
ALD	–79.4 ± 1.9	0.31	–27.9 ± 1.1	0.37	–21.9 ± [1.6–3.5]^b^
					–25.0 ± 0.5 (0.37)^c^
					≈ −70^d^
					–8.7 ± 1.0^e^
ADE	–89.9 ± 1.6	0.28	–35.0 ± 0.4	0.33	–30.6 (0.33)^f^, 131.4^g^, −35.4 ± 1.8^h^
CYT	–70.2 ± 1.3	0.28	–29.4 ± 0.6	0.32	–30.0 (0.29)^f^, 131.8^g^, −18.5 ± 1.0^h^
GUA	–104.5 ± 2.5	0.28	–40.6 ± 0.4	0.33	–40.7 (0.33)^f^, 142.3^g^, −34.6 ± 1.1^h^
THY	–78.7 ± 2.4	0.28	–31.1 ± 0.6	0.32	–20.1 (0.35)^f^, 111.7^g^, −18.3 ± 0.5^h^
PYR	–38.3 ± 0.7	0.28	–18.3 ± 0.3	0.33	
DOX	–135.0 ± 5.6	0.34	–79.6 ± 2.3	0.44	

a Standard deviations obtained from the bootstrap analysis and reference values taken from the literature are also listed.

b Value obtained through thermodynamic integration in ref (60).

c Values obtained through metadynamics in ref (61).

d Values obtained through well-tempered metadynamics in ref (62).

e Values obtained through well-tempered metadynamics in ref (63).

f Values obtained through well-tempered metadynamics in ref (64). Numbers in parentheses refer to reported minimum distances.

g Values obtained in ref (65).

h Values obtained through well-tempered metadynamics in ref (14).

### 3.1 Alanine Dipeptide for Validation

Since amino acids constitute the building blocks of proteins, their adsorption on metal surfaces has been widely studied in recent years.^66−68^ In particular, capped amino acids have been employed as model structures to mimic the behavior of longer peptides.^60,62,69^ For this reason, we selected alanine dipeptide to test our computational strategy against previously published computational results.

**Figure 4:**
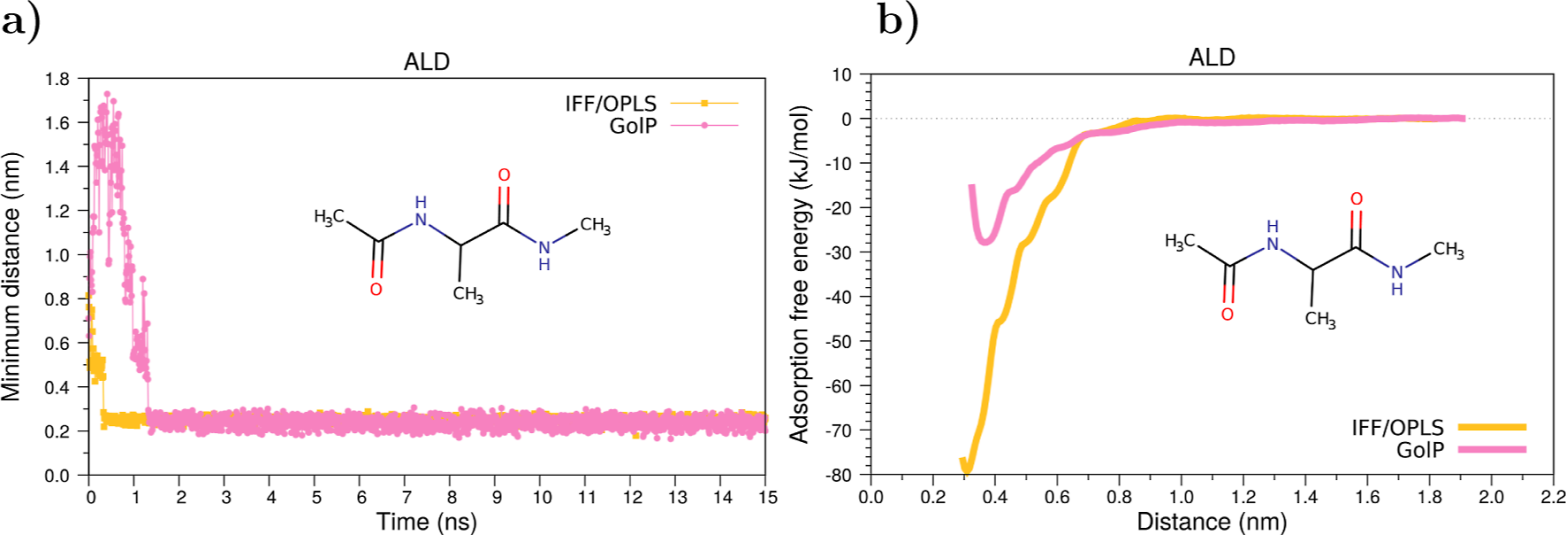
(a) Evolution of the time of the minimum distance and (b) free energy profiles along the reaction coordinate for the adsorption of the alanine dipeptide on a gold surface. FF: IFF/OPLS and GolP.

Concerning the adsorption dynamics, we plotted in Figure 4a the time evolution of the minimum distance of ALD parametrized with OPLS/AA. The corresponding plots using the CHARMM FF are given in the Supporting Information (Figure S1). Figure 4a shows that ALD rapidly approaches the gold surface and, once adsorbed, it remains bound to it, mainly with the methyl lateral group pointing up toward the bulk water in agreement with descriptions of previous works.^38^ Exceptionally, in the GolP-CHARMM case (see Figure S1 in Supporting Information), ALD desorbs a couple of times, suggesting that there is a competitive effect for the interactions with the water molecules in the vicinity and with the gold surface; this behavior is expected, being a known feature of this FF.^40^ It can be noticed that the structural disposition of the adsorbate allows for a more flat orientation that favors the contact of the majority of its atoms, especially heteroatoms, with the gold surface.^38,63^ Even when ALD desorbs in some intervals of the MD run, it then returns to the metal in the same orientation as before. It is worth mentioning that our computed minimum distance agrees better with the simulation works in the GolP case (0.37 nm in Table 1), and it is shorter (0.31 nm) when IFF is used to describe the gold surface. Despite having found longer minimum distances with GolP FFs, there are no water layers between the molecule and the surface in the adsorbed state.

Now we move on to comment on the outcomes of the PMF profiles obtained by the US technique. The overlapping of the umbrella windows for the GolP case is shown as a set of histograms in Figure S2 in the Supporting Information. As highlighted in Table 1, our results on adsorption free energy are in line with other works. The value obtained by using IFF (−79.4 kJ/mol) agrees with ≈−70 kJ/mol reported by Shao and Hall^62^ from well-tempered metadynamics calculations on gold nanoparticles of varying size. Discrepancies may arise from the use of a different FF to treat ALD (GROMOS vs OPLS/AA), from the mobility of gold atoms (the authors of ref (62) kept the positions of gold atoms fixed), or from the specific facet involved in the binding process. GolP results can be compared with those provided by Hoefling et al.^60^ and Bellucci and Corni,^61^ who used two diverse pathway-based methods to compute adsorption free energies for alanine dipeptide. Clearly, the agreement is good in terms of both minimum distance and adsorption free energy. In the case of GolP-CHARMM, the value is similar to the one reported in ref (63).

By looking at the shape of the free energy profiles in Figure 4b, we note that OPLS-derived profiles are smooth and do not present evident barriers for adsorption. In contrast, those profiles coming from CHARMM FF, in particular from GolP-CHARMM (Figures S1 and S3 in Supporting Information), exhibit a small barrier of 2.3 kJ/mol around 0.5 nm; similarly to ref (63), energy barriers are more pronounced and better evidence the three steps of the adsorption process.^60^ In general, our data indicate that the adsorption process of ALD on gold is very sensitive to the FFs used to describe the surface and the molecule; adsorption free energy values can range from −5.3 to −79.4 kJ/mol, as can be seen in the bottom panel of Figure 3. Notwithstanding, the assessment of such values requires a direct comparison with experimental data that, to the best of our knowledge, are not available in the literature. A more in-depth discussion of the diverse performance of the FFs employed here is given below.

For the sake of completeness, we performed additional MD and US simulations to evaluate the effect of gold polarization within the IFF model (pol IFF) on the adsorption dynamics and energetics of the alanine dipeptide. The results, which are depicted in Figure S4 in the Supporting Information, show that the inclusion of polarization effects within IFF leads to negligible differences in both minimum distances and adsorption free energy profiles, regardless of the usage of OPLS/AA or CHARMM FF to describe the molecule.

### 3.2 Performance of FFs: Nucleobases

The biomolecular adsorption of nucleobases on nanoscale surfaces, especially concerning gold, is a mature topic at present. Studies range from DFT calculations^70,71^ to classical MD simulations that can include enhanced sampling techniques.^64,65,72,73^ We assessed how the description of the DNA bases and the gold provides diverse nuances in the understanding of the adsorption process. Regardless of the initial orientation, after adsorption, every nucleobase lies parallel to the surface (horizontal, slightly tilted orientation), as can be seen in panel b of Figure 2, and in line with the configurations reported by Rosa et al.^64^ In these preferred adsorption geometries, molecule-surface distances from Tables 1 and S1 are around 0.28 and 0.33 nm and have almost the same value independently of the nucleobase, allowing no water molecules in between, similarly to ALD. A comparison of the minimum distances in Figure 3 evinces that nucleobases adsorb at longer distances from the Au(111) surface when GolP and GolP-CHARMM are used. In addition, there is a fair agreement with distances reported by previous works (0.29–0.35 nm).^64,72^ Plots for the evolution of the minimum distances along the trajectory are shown in Figure S5 in Supporting Information. As a general rule, we found that once the purine or pyrimidine is adsorbed on the surface, it does not desorb during the entire MD simulation, implying a preference for the adsorbed configuration in agreement with previous works,^73^ except for ADE and CYT in GolP-CHARMM, which adsorb and desorb multiple times. Also, Figure S5 highlights an important feature related to two possible accommodations of the nucleobases: one at the *d*_min_ written in Tables 1 and S1 and another one at a longer distance, ≈0.5 nm, where each nucleobase is adsorbed with the plane of the molecule in a parallel orientation with respect to the substrate surface but with solvent molecules mediating the interaction. An example of both configurations is included in panels e and f in Figure 6 (see below). Notice that all of the data listed in Tables 1 and S1 are associated with the most favorable conditions (orientation and distance) for each interface.

Experimental studies providing structural information on nucleobases adsorbed to surface interactions are currently limited, and in the past, findings were based on the study of the changes of vibrational modes and supported a certain extent of tilt.^74^ Tilted orientations were also found in several simulations using MD.^64^ Concerning energies, we refer to values obtained from two indirect experimental techniques. First, ref (14) reports the following inferred AFEs for the nucleobases: ADE = 36 ± 11 kJ/mol, GUA = 29 ± 3, CYT = 28 ± 2, and THY = 21 ± 7 kJ/mol from SMFS experiments that measure the desorption force of the four ssDNA oligomers from the aqueous Au(111) interface. Second, there are available values of heats of desorption from temperature-programmed desorption (TPD) experiments, which detail a G > A > C > T trend for DNA monolayer bases acting as adsorbates on gold,^75^ but carried out in dried samples. The same trend was then confirmed in the experimental work of ref (74). Computational works by Rosa et al.^64^ and Rapino and Zerbetto^65^ reproduced the trend (ref (65) also replicates the values in the gas phase) employing computations by means of well-tempered metadynamics or calculating adsorption free energies from the energies of the optimized cell and fragments (Au surface and molecule), respectively. Data in Table 1 shows that with GolP/OPLS, we obtained AFE values similar to those reported in refs (14) and (64), except for THY, which is no longer the nucleobase that interacts more weakly with the gold surface. Indeed, that is the reason our trend differs from the experimentally reported one.

Moreover, Table 1 shows that when the gold surface is described by the IFF, larger adsorption energies are found and thus more similar values to desorption enthalpies in ref (65) and in vacuo free energies in ref (64). This observation again highlights the fact that the level of treatment for the solvent and the inclusion of dynamic/entropic effects might eventually alter the individual values of the AFE. However, it is difficult to establish whether there is a systematic overestimation of these IFF-calculated adsorption energies in the absence of experimental data for the adsorption of single ADE, CYT, GUA, and THY molecules on hydrated Au(111).

**Figure 5:**
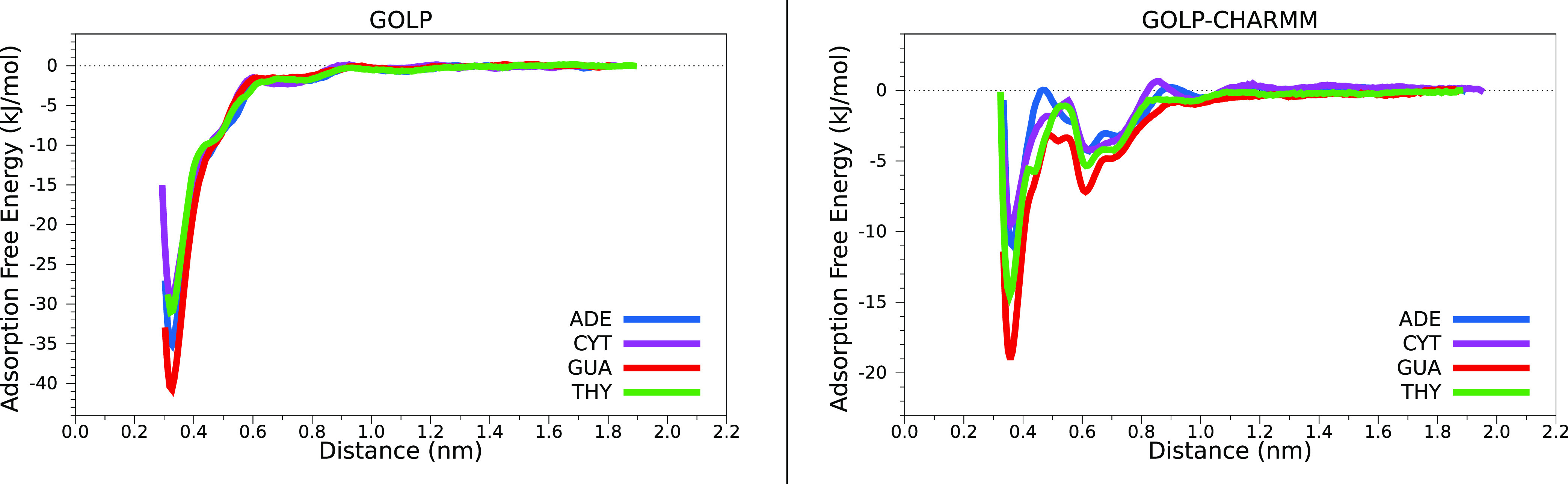
Adsorption free energy profiles of the four nucleobases modeled with OPLS/AA on the left and CHARMM on the right by using GolP FFs.

**Figure 6:**
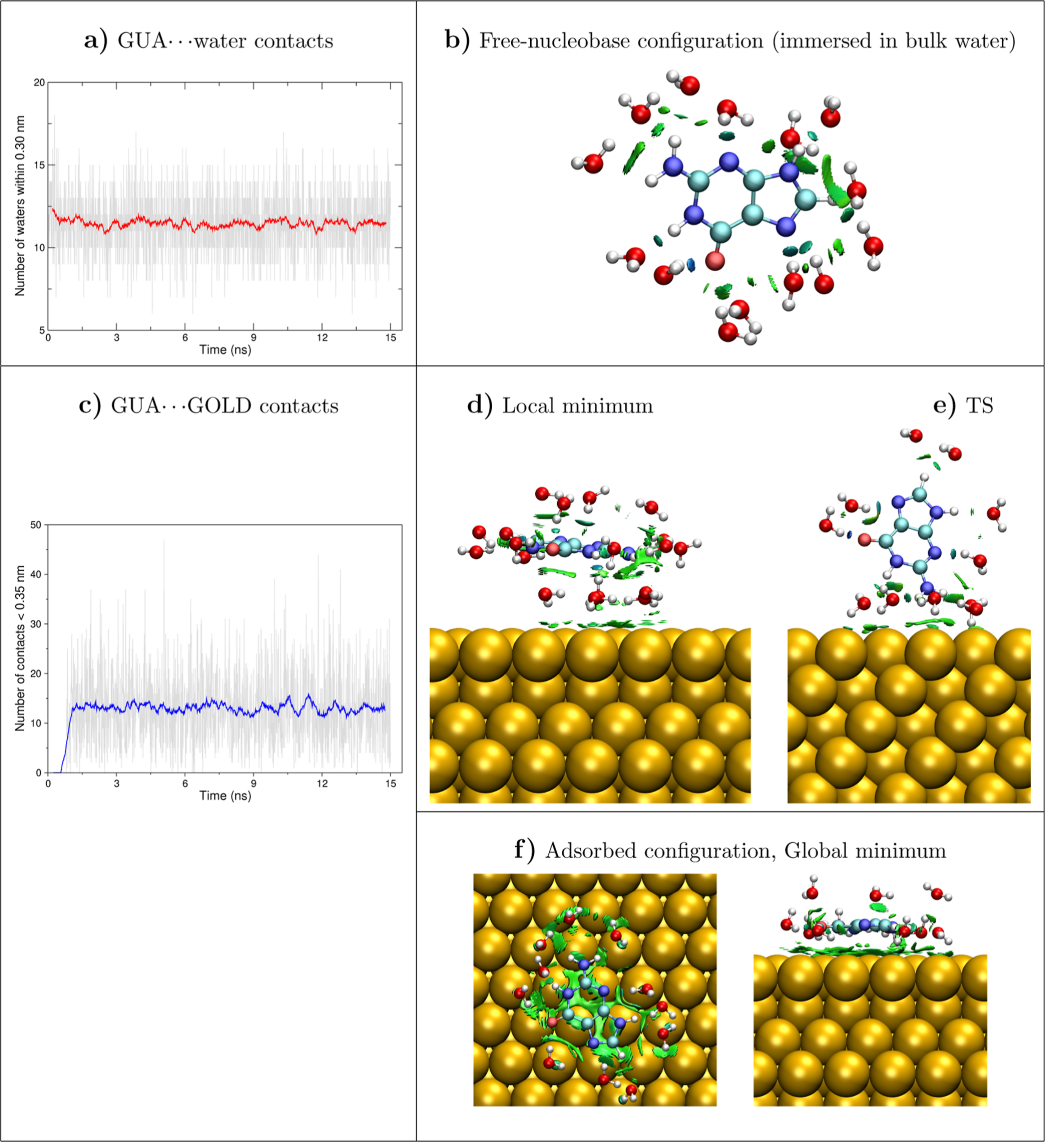
Analysis of the interactions occurring during the adsorption of guanine on the gold surface (GolP-CHARMM FF). Left: evolution in time of the number of contacts with the surface and water molecules surrounding guanine. Right: NCI plots of guanine in its in-solution state, local minimum, transition state, and global minimum (top and lateral view) configurations. Water molecules within 0.3 nm of guanine are shown. Similar plots using the IFF/CHARMM FF are displayed in Figure S10 in the Supporting Information.

Figure S6 reports the free energy landscape for the formation of an interface between each nucleobase and Au(111) in a water solution as a function of the nucleobase COM-surface distance. To compare the energy profiles obtained for the four nucleobases with the different FFs, curves are plotted together in Figures 5 and S7 for the GolP family FFs (GolP and GolP-CHARMM) and IFF, respectively. From these results, it is clear that OPLS gives rise to the G > A > T > C trend, whereas CHARMM provides G > T > A > C, both orderings differing from that of the experimental propensity to desorb.^75^ Beyond the sensitivity of the AFE values to the different FF mixtures, the two panels in Figure 5 illustrate the existence of lowest-free-energy global minima in all cases (for GUA it is displayed in Figure 6f) with their corresponding equivalent equilibrium distances, 0.28 and 0.33 nm, when GolP/OPLS and GolP/CHARMM are used to treat the surface/molecule, respectively.

When looking at the energetics of the whole adsorption process, we can recognize the presence of a second minimum in each profile (for GUA, it is displayed in Figure 6d for GolP-CHARMM and Figure S10d for IFF-CHARMM), holding a water layer between the gold substrate and the adsorbate and located in the 0.50–0.70 nm interval from the surface as suggested by the plots of the minimum distances (Figure S5 in Supporting Information). These two minima are connected by a transition state (TS). For GUA, in the GolP-CHARMM case, the TS is characterized by the NH_2_ group pointing toward the surface and several water molecules located at the same distance from the metal, as displayed in Figure 6, panel e. In the IFF-CHARMM case, GUA directly interacts with gold atoms through the hydrogen linked to atom C8 (see the numbering in Figure 1).

The energy barriers are exacerbated in the CHARMM-derived profiles, as can be seen in the right panel of Figure 5. The appearance of TS configurations agrees with the reported findings when computing the free energy of the interaction of nucleobases on Au using PMF in conjunction with the GolDNA-AMBER force field.^73^ According to the authors, the barrier exists because of a water displacement from the interface in the competing process, at that distance, between the adsorbate and the solvent for the surface, in line with what we found here for the nucleobases, which must displace the water molecules before getting closer to the gold substrate.

The DNA base interactions on the gold surface and with the water molecules are further analyzed in Figures 6 and S10, which display the number of contacts and the noncovalent interaction (NCI) plots for GUA while getting adsorbed on the surface. According to Figure 6, panel a, the number of waters at the beginning of the trajectory is slightly higher since the adsorption process reveals that the molecule spent some time immersed in bulk water before being adsorbed just above the surface. However, there are always at least 11 waters in the vicinity that keep interactions with the nucleobase. Such interactions are confirmed by the blue lentils and small green NCI surfaces that appear in Figure 6, panel b, to indicate the formation of hydrogen bonds (HB) with the solvent. The maximization of the GUA···water contacts when the molecule is far from the gold coincides with the null number of interactions with the metal (see the curve in the first ns in Figure 6, panel c. NCI surfaces for GUA in its minima (local and global) and its TS are plotted in panels d,e of Figure 6. It can be noticed that the energetic difference between both minima is related to the green NCI (attractive) surfaces that are more spread when GUA is adsorbed onto the surface and follow the shape of the aromatic rings just after adsorption. In the latter position, there are up to 14 atom pairs that fall within 0.35 nm, a threshold chosen based on the minimum distance plots of Figure 3. When performing the same comprehensive analysis for GUA/gold/water using the IFF-CHARMM combination, we observed notable differences with respect to the GolP-CHARMM adsorption pathway. Specifically, IFF-CHARMM resulted in a higher number of GUA/gold contacts compared to the GolP-CHARMM-adsorbed configuration, as highlighted in Figure S10 panel c. This increased interaction between guanine and the gold surface is reflected in a 2-fold value of the NCI integral in the global minimum, as outlined in Table S2. By further comparing the integral values for the NCI surfaces reported in Table S2, we note that GolP-CHARMM gives, in contrast, a stronger interaction with the solvent when guanine is present in the bulk.

### 3.3 Further Applications: Nonstandard Ligands

To showcase the applicability of our methodology, we performed a complete analysis of two nonstandard ligands, pyridine and doxorubicin, that have been widely used in surface enhanced Raman scattering (SERS) experiments.^76−87^

As expected, both PYR and DOX, whenever adsorbed, assume an orientation with the aromatic rings lying almost parallel to the surface, likewise the nucleobases. Clustering analysis for DOX in Figure S8 in Supporting Information shows that irrespectively of the FF, there are around five structural families, whose representative structures differ from each other in the flexibility of the anchor (substituent in the anthraquinone ring) and the daunosamine, the latter known to be involved in the groove binding when DOX is intercalated into DNA. This flexibility has already been reported in other works for free, solvated, and intercalated DOX.^88−91^ As a matter of fact and unlike previous MD for DOX,^89,92,93^ with the IFF/OPLS combination, one of the hydroxyl groups attached to the ring C (see Figure S8) rotates during the MD simulation, breaking the standard strong intramolecular interaction and being oriented toward the oxygen atom that bridges the sugar group, forming a new O–H···O HB.

**Figure 7:**
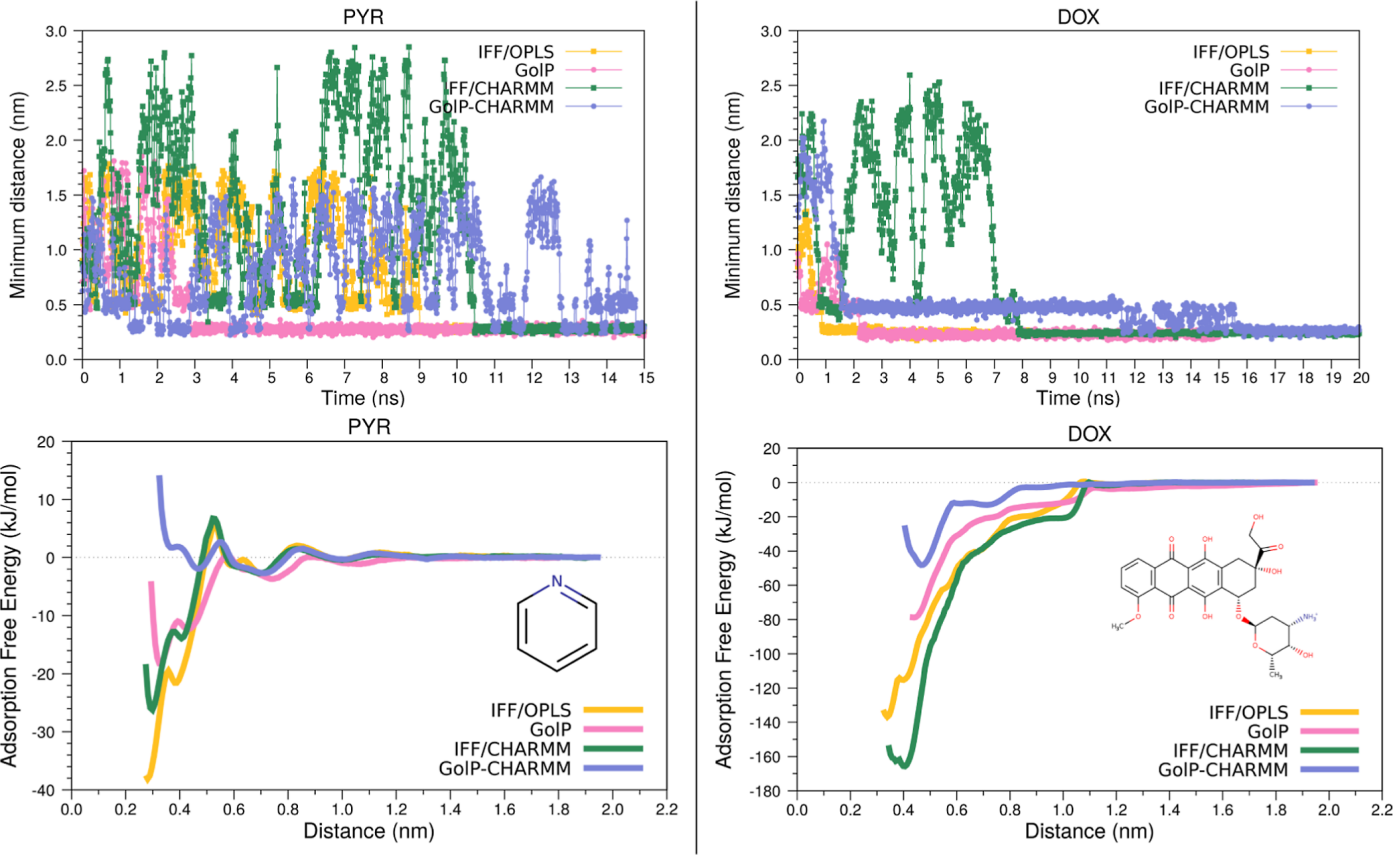
Minimum distance and adsorption free energy plots for PYR (left) and DOX (right) for all of the possible combinations of force fields.

The evolution of minimum distance molecule···surface and AFE profiles is displayed in Figure 7. Minimum distances for PYR have the same value, 0.28 nm, when IFF is used, regardless of the FF employed to describe the molecule, while GolP-derived distances are longer, as reported in Tables 1 and S1. Longer distances are also seen for DOX: GolP parametrization allows the molecule to stay up to 1 Å farther (0.34 vs 0.44 nm in Figure 3) with respect to the gold surface than IFF one, even if there are no water molecules mediating the interactions with gold atoms.

As can be appreciated in the right panel in Figure 7, when GolP-CHARMM is used, there is a jump resulting in a reduction of the minimum distance, a feature shared also with the other adsorbates. In fact, at around 0.5 nm from the gold surface, i.e., before jumping to the final adsorbed configuration, there seems to be an initial favorable position for DOX, in which the daunosamine portion is located, such that it diminishes the possible steric hindrance for the rings to be positioned parallel to the substrate. At that distance, some water molecules surround DOX, mediating the interaction with the gold surface, as shown in the NCI plot in Figure S13 in Supporting Information.

In contrast to the permanent adsorption of DOX, the time evolution of PYR···Au minimum distances in Figure 7, left panel, highlights the difficulty of this molecule in adsorbing and remaining adsorbed, which is further underlined by the corresponding adsorption free energy profiles. PYR AFE profiles exhibit diverse energy barriers, suggesting, in turn, different transition states. For example, in the IFF cases, one of these energy barriers, with ≈5.7 kJ/mol, lies above the zero reference. Then, pyridine in the transition state is perpendicular with respect to gold (around 90°, better between 60 and 120°), and in the umbrella window, the nitrogen atom stays most of the time directly above gold atoms. In spite of the barriers, the adsorption process still occurs for IFF/OPLS, IFF/CHARMM, and GolP, as opposed to the GolP-CHARMM AFE profile, where there are shallow minima (the lowest-energy one has a −2.13 kJ/mol value in Table S1). In addition, the GolP-CHARMM PMF curve is almost entirely above the zero reference, thus confirming the tendency of PYR to remain in bulk water.

Concerning DOX, as in the previous cases, adsorption free energy values are found to be very sensitive to the combination of the FFs, up to 4-fold different (−47.6 vs −163.1 kJ/mol). By looking at Figure 3, we notice that DOX is the only adsorbate that alters the trend of the predicted adsorption free energies with the tested FF combinations, which we find to be in absolute values AFE_IFF/OPLS_ > AFE_IFF/CHARMM_ > AFE_GolP_ > AFE_GolP-CHARMM_. We speculate that since DOX is a medium-sized, more complex molecule than the other adsorbates studied here, the different sorting for the AFE values could be related to the diverse geometries that DOX presents in the minimum of the profiles. Figure S9 superimposes DOX structures in the adsorbed configurations with the four FFs. It is clear that the DOX structure comes from the combinations that change the trend, i.e., IFF/OPLS and IFF/CHARMM are those that are more bent, and in particular, IFF/OPLS (in goldenrod) exhibits the hydroxyl group rotation mentioned above.

### 3.4 Discussion

So far, we have seen that structural properties and AFE values are quite sensitive to the FF choice. One of the desirable features of any FF is that it should be system-independent, and indeed, our results are an attempt to contribute to the answer to the ever-open question in the literature about what FF to employ when modeling molecule-surface interactions. In this respect, there are a few approaches: (i) specifically parametrize interfacial FFs to describe different molecular moieties on gold surfaces; (ii) take a FF rigorously parametrized to tune specific interfacial interactions (e.g, GolP and GolP-CHARMM) and use it as it is; or (iii) combine existing FFs for the different components of the system (e.g., IFF/CHARMM). Surely, the former strategy is the most expensive but also accurate for describing the specific interaction occurring at the interface.^17,19,35,94,95^

Resorting to the transferability principle, in our protocol, we have used the second and third options, and from our findings summarized in Figure 3, the strength of the adsorption is affected by the FFs chosen for both the molecule and the gold, but it seems that the FF of the molecule determines the shape of the profile, so the adsorption pathway and relative trends of affinity (see nucleobases case in Figure 5), while the FF for treating the surface more strongly determines the minimum distance molecule···Au and thus the absolute AFE values. In particular, the GolP-CHARMM combination returns detailed shapes of the profiles, e.g., energy barriers, whereas IFF in any combination permits the molecule to get closer to the gold surface and to adsorb faster and stronger.

In the end, the selection of one of the three above-mentioned options is conditioned by the details that one is interested in exploring. For example, concerning an application in the sensing field, the most interesting things are whether the molecule remains adsorbed along the time or not, the molecule-surface distance, and the orientation. Therefore, a FF combination like GolP/OPLS could be a good balance between cost-effectiveness and accuracy in providing such information.

It is worth mentioning the role of the solvent as a third component of the interface. Water mediates molecule-surface interactions, and its accurate treatment could influence the final results. The selection becomes challenging since the FF used to represent water molecules must be coupled to those for the adsorbate and the surface. We used SPC and TIP3P FFs for OPLS and CHARMM, respectively. The use of OPLS/SPC and CHARMM/TIP3P is a common practice when simulating organic molecules, peptides, and biomolecules, and both have been proven to afford reliable results in interfacial cases with metals.^16,41^ However, the combination of OPLS/TIP3P can in principle be used and, in our case, can lead to non-negligible differences in adsorption free energies, whose reliability should be checked and validated.^95^

To unravel the influence of the FF used to model the solvent, we performed further MDs for water/gold, representing the situation without molecular adsorption. Our analyses, supported by NCI indexes listed in Table S3 and displayed in Figures S11 and S12, suggest that TIP3P water molecules engage in intensified competition with ligands for adsorption on the surface, thereby giving rise to more dynamic phenomena. In fact, the greater affinity of water molecules for gold when modeled with the TIP3P FF results in a more dense hydration layer over the Au(111) surface, regardless of the FF employed to describe it, which is clearly visible when examining Figures S11 and S12. As a consequence, this competition challenges the entry of adsorbates across the hydration layer to reach the surface. Conversely, in the case of SPC water models, the adsorbed molecule finds it comparatively easier to reach the metal surface, even though water molecules are in closer proximity to the surface.

The interaction strength between the water layer and the gold surface, as determined by the combination of water/surface force fields (FFs), significantly influences the predicted AFE values of the adsorbed molecules. We have consistently observed a trend wherein SPC water models yield stronger AFE values compared to TIP3P when using the same FF for gold. Importantly, as shown in Figures S11 and S12, the use of IFF for gold surfaces results in a higher number of water molecules (ca. 200 vs 160) at the same threshold value of 0.35 nm. Additionally, IFF affords close proximity between the adsorbate and the gold surface, as highlighted in the plot of Figure S10c. Ultimately, these proximal contacts, water···gold and adsorbate···gold, enhance the overall affinity.

Hence, our findings, as summarized in Figure 3, suggest that distinct water models result in varying binding affinities, and these disparities are further accentuated by the selection of force fields used for the ligand and the surface.

## 4 Conclusions

In this work, we have studied the adsorption process and the absolute and relative binding affinities of seven adsorbates, namely, alanine dipeptide, the four DNA nucleobases, and a couple of nonstandard ligands, pyridine and doxorubicin, on the Au(111) surface. Notably, an accurate description of the system’s components depends on the choice of force fields for the molecule, solvent, and gold substrate. Hence, in order to understand the role played by the parametrization of the molecule-surface interactions, we have carried out a comparison between different combinations of FFs: IFF/OPLS, IFF/CHARMM, GolP/OPLS, and GolP-CHARMM.

Our classical MD simulations provide valuable insight into these complex systems’ dynamic features, thanks to an effective sampling of the phase space of the molecule in close proximity to the nanosurface while also accounting for solvation effects. The AFE values, calculated by means of an umbrella sampling procedure, indicate that for the entire set of molecules, adsorption on gold is favorable, with pyridine and doxorubicin having the lowest and highest affinities, respectively, although the values depend on the FF combination used. Minimum molecule-surface distances revealed that guanine is the adsorbate that gets closer to Au atoms, but in general, *d*_min_ < 0.44 nm for all molecules. Despite these apparently long distances and the slightly tilted orientations, in the adsorbed configurations, there are no water molecules mediating the molecule-surface interactions in each studied system. Concerning the adsorption pathway, each FF combination leads to different results that have to be interpreted based on the final scope. For example, if there is any interest in the sensing field, GolP/OPLS offers information more in agreement with that available in both experimental and computational works, in terms of both AFE values and minimum distances. On the other hand, GolP-CHARMM gives more detailed free energy profiles that present energy barriers and transition state configurations.

After monitoring the trajectories, there is a switch in the interactions that take place: first, molecule/water when the molecule is immersed in the bulk solution far from the surface, then molecule/water/gold in the transition state with a layer of water between, and then water/molecule/gold with the molecule in direct contact with the surface. All these interfaces are the result of a series of hydrogen bonds and weak van der Waals interactions that appeared as noncovalent interaction surfaces when characterized by NCI plots. The eventual formation of covalent bonds between Au and N atoms is out of the scope of this work and should be analyzed with reactive FFs.^96−99^

Lastly, our computational protocol allows collecting information on the distances, orientations, and energetics of molecules with respect to a nanosurface that is essential in view of subsequent spectroscopic applications, such as SERS. In addition, the in silico design of plasmonic sensors and the foreseen extension of the modeling to the simulation of spectral signals may lead to an improvement in the performance of detection techniques for small molecules. Indeed, we plan to investigate that in future works.

## Data Availability

All adsorbate structures have been optimized by using the AMS code (version 2020.202, http://www.scm.com). Gold slabs have been generated using the atomic simulation environment (ASE) Python module (https://wiki.fysik.dtu.dk/ase/). MD simulations and umbrella sampling calculations of the different adsorbate/water/gold nanosurfaces have been performed by using Gromacs version 2020.4 (https://www.gromacs.org). NCI calculations have been performed by using NCIPLOT4 (https://www.lct.jussieu.fr/pagesperso/contrera/nciplot.html).

## Supporting Information Available

Adsorption free energies and minimum distances of the seven studied molecules with IFF/CHARMM and GolP-CHARMM; plots of minimum distances and adsorption free energy profiles for alanine dipeptide and the four nucleobases; NCI analyses; and clustered structures of doxorubicin (PDF)
