# Supplementary material for: Computational Insights into the Adsorption of Ligands on Gold Nanosurfaces
Source: J Phys Chem A. 2023 Nov 22;127(48):10282–94. doi: 10.1021/acs.jpca.3c05560 (PMC10711798; doi:10.1021/acs.jpca.3c05560)
Supplement: Supplementary file 1 — jp3c05560_si_001.pdf [file jp3c05560_si_001.pdf]

Computational Insights into the Adsorption of Ligands on Gold Nanosurfaces

Sveva Sodomaco,† Sara Gómez,† Tommaso Giovannini,† and Chiara Cappelli,†

†*Scuola Normale Superiore, Classe di Scienze, Piazza dei Cavalieri 7, 56126, Pisa, Italy*

Correspondence: sara.gomezmaya@sns.it, chiara.cappelli@sns.it

Supplementary information

Contents

1 General comparisons **S2**

2 Alanine dipeptide **S3**

2.1 Analyses S3

2.2 Non-polarizable vs polarizable IFF S5

3 Nucleobases **S6**

3.1 Minimum distances S6

3.2 Profiles S7

3.3 Comparison between profiles with IFF S8

4 Doxorubicin **S9**

5 NCI analyses **S11**

1 General comparisons

Table S1: Computed average adsorption free energies ΔE_{ads} and minimum distances d_{min} for adsorbed molecule/Au(111) systems of Figure 1, using CHARMM for each molecule and IFF and GolP-CHARMM for the gold surface. Standard deviations obtained from bootstrap analysis are also reported. Reference values taken from the literature are listed as well.

LIGAND	IFF		GolP-CHARMM		Other works
	ΔE_{ads} (kJ/mol)	d_{min} (nm)	ΔE_{ads} (kJ/mol)	d_{min} (nm)	
ALD	-50.9 ± 1.2	0.30	-5.3 ± 0.9	0.35	-21.9 ± [1.6-3.5][a]<br>-25.0 ± 0.5 (0.37)[b]<br>≈ -70[c]<br>-8.7 ± 1.0[d]
ADE	-66.5 ± 1.7	0.28	-11.0 ± 0.4	0.33	-30.6 (0.33)[e], 131.4[f], -35.4±1.8[g]
CYT	-63.2 ± 0.6	0.27	-9.4 ± 0.4	0.33	-30.0 (0.29)[e], 131.8[f], -18.5±1.0[g]
GUA	-90.0 ± 1.6	0.26	-19.1 ± 0.5	0.33	-40.7 (0.33)[e], 142.3[f], -34.6±1.1[g]
THY	-78.9 ± 1.4	0.27	-14.5 ± 0.4	0.33	-20.1 (0.35)[e], 111.7[f], -18.3±0.5[g]
PYR	-26.1 ± 0.4	0.28	-2.13 ± 0.7	0.35	
DOX	-163.1 ± 3.22	0.32	-47.6 ± 2.0	0.42	

[a]Value obtained through thermodynamic integration in Ref. 1
[b]Values obtained through metadynamics in Ref. 2
[c]Values obtained through well-tempered metadynamics in Ref. 3
[d]Values obtained through well-tempered metadynamics in Ref. 4
[e]Values obtained through well-tempered metadynamics in Ref. 5. Numbers in parentheses refer to reported minimum distances
[f]Values obtained in Ref. 6
[g]Values obtained through well-tempered metadynamics in Ref. 7

2 Alanine dipeptide

2.1 Analyses

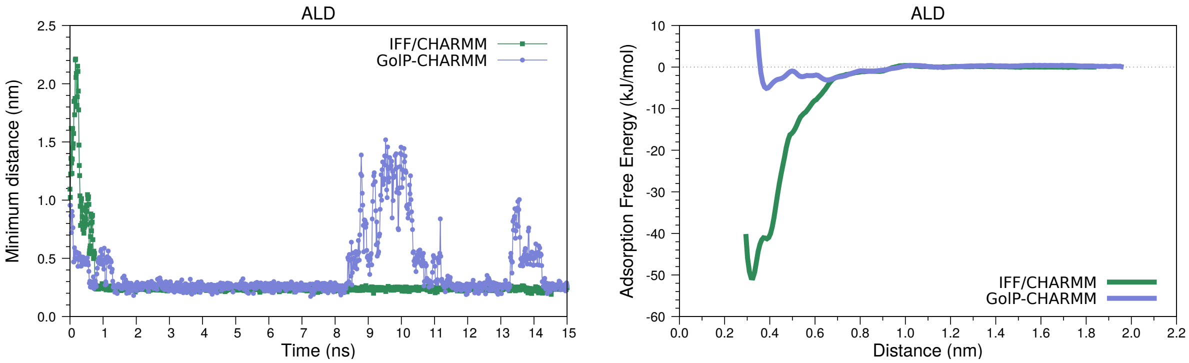

Figure S1: Evolution on time of the minimum distance of alanine dipeptide from the gold surface (left) and free energy profiles along the reaction coordinate (right) for the cases IFF/CHARMM and GolP-CHARMM.

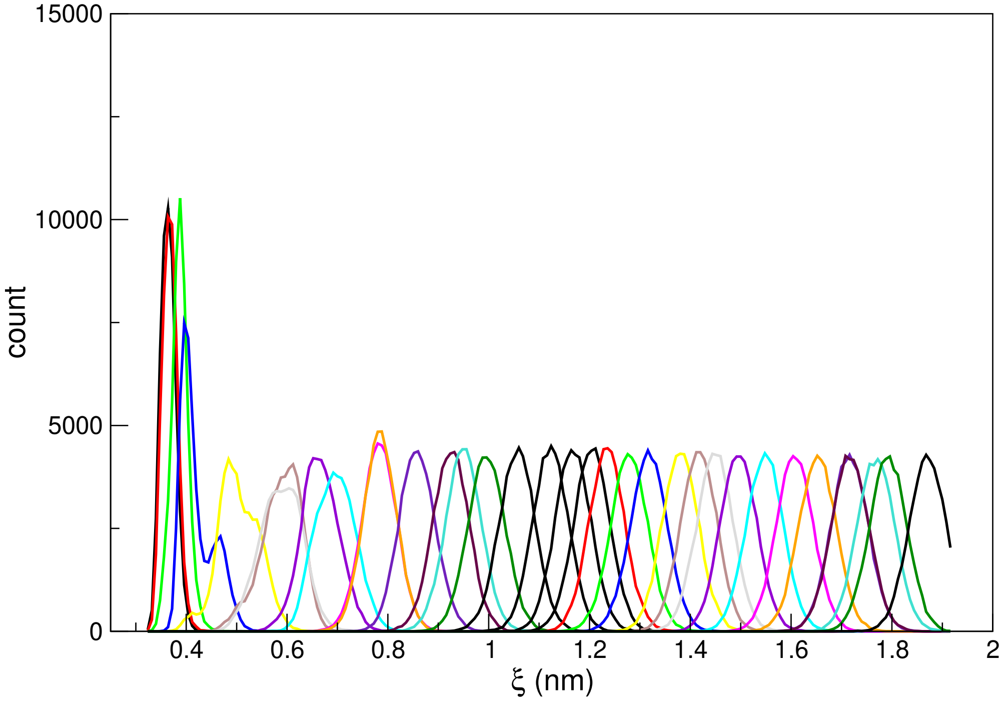

Figure S2: Histograms for the umbrella windows used to sample the reaction coordinate for the adsorption of the alanine dipeptide. FF: GolP

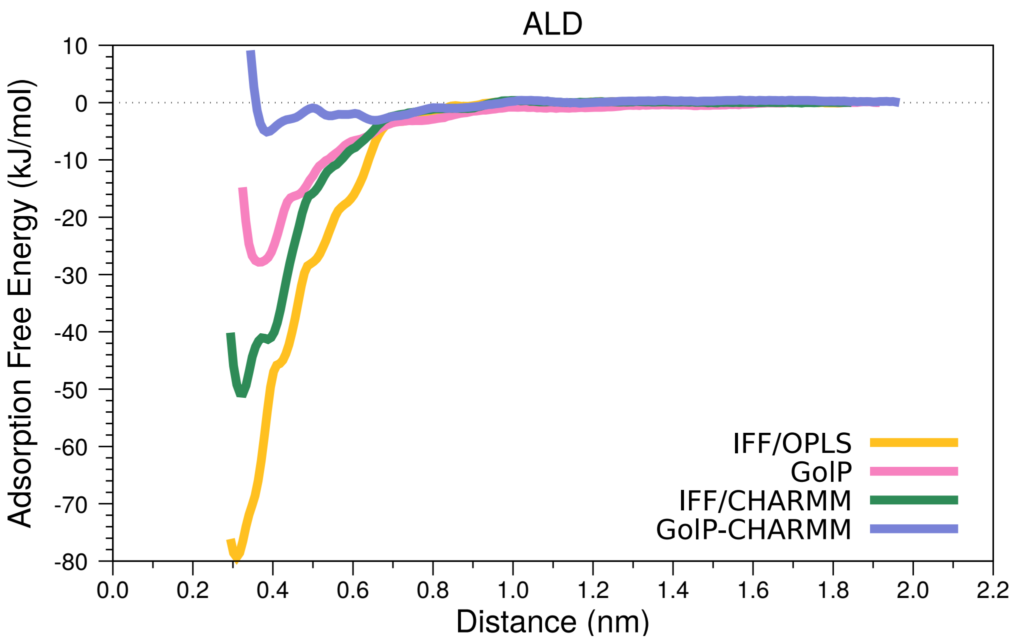

Figure S3: Comparison of the free energy profiles for the adsorption process of alanine dipeptide on gold, computed with the four combinations of FFs.

2.2 Non-polarizable vs polarizable IFF

(a) OPLS/AA

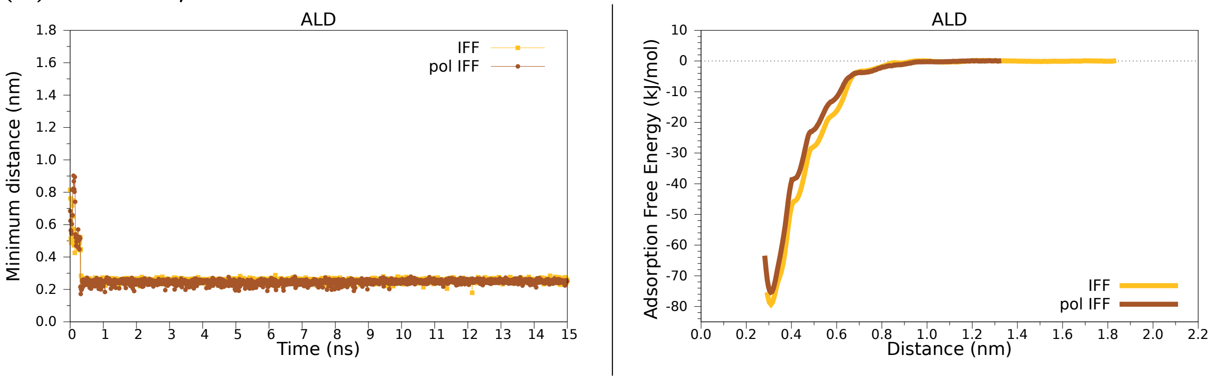

(b) CHARMM

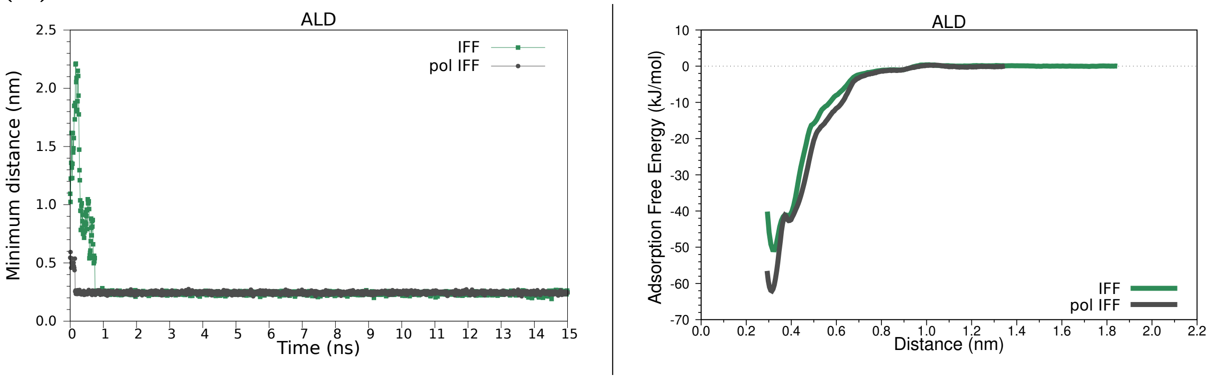

Figure S4: Evolution of the minimum distance (left) and free energy profiles (right) in the a) OPLS/AA and b) CHARMM case.

3 Nucleobases

3.1 Minimum distances

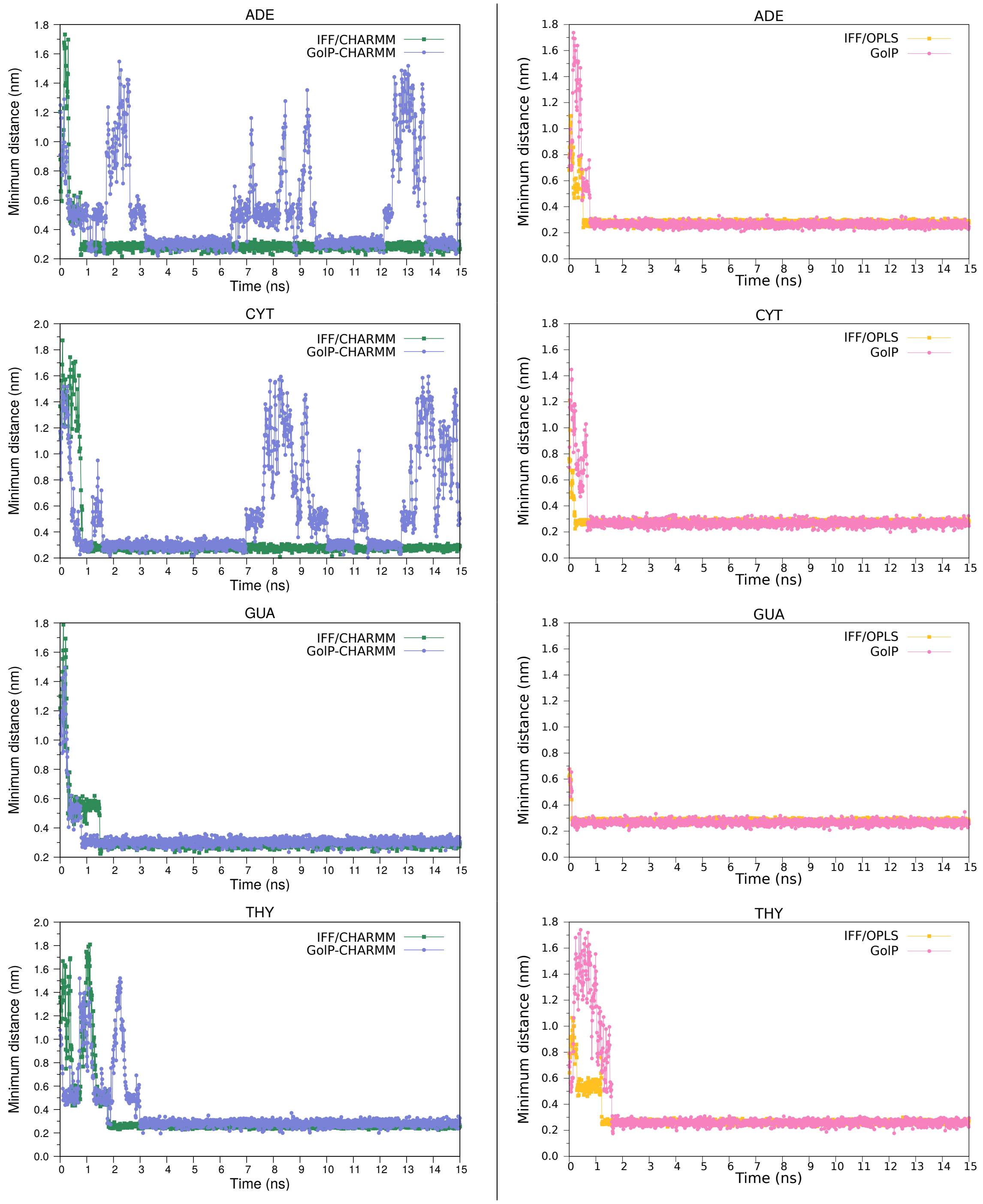

Figure S5: Left column: minimum distance plots for each nucleobase employing OPLS/AA. Right column: minimum distance plots for each nucleobase employing CHARMM.

3.2 Profiles

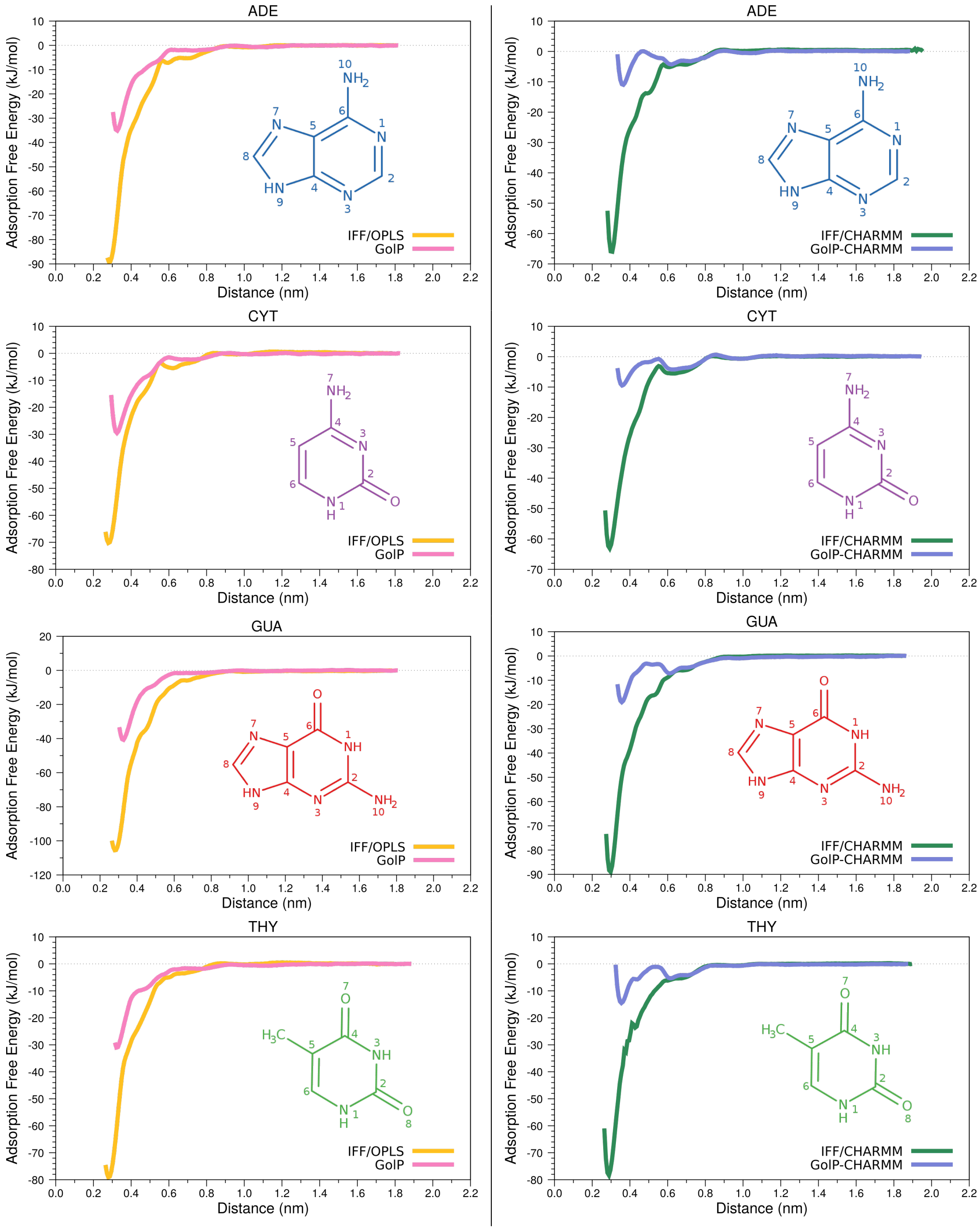

Figure S6: Left column: adsorption free energy profiles for each nucleobase employing OPLS/AA. Right column: adsorption free energy profiles for each nucleobase employing CHARMM.

3.3 Comparison between profiles with IFF

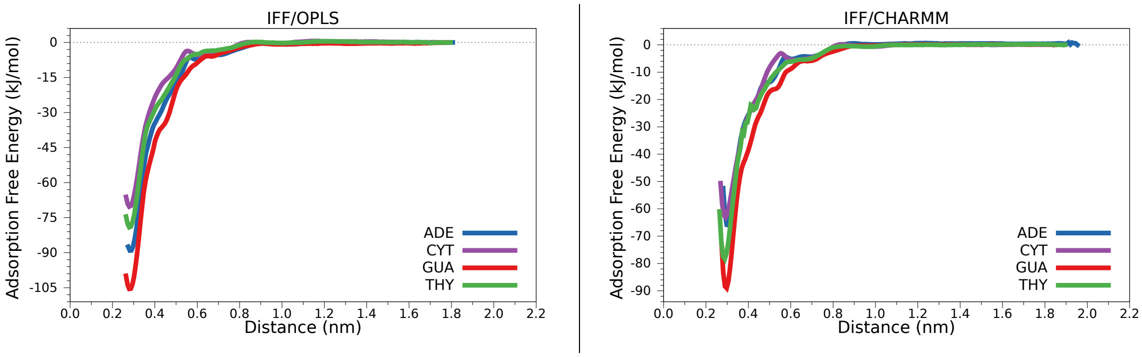

Figure S7: Adsorption free energy profiles of the four nucleobases modeled with OPLS/AA on the left and CHARMM on the right using IFF FF.

4 Doxorubicin

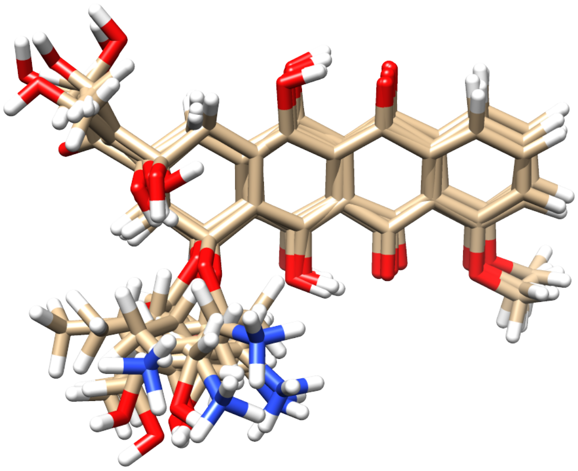

Figure S8: Superimposed representative structures from clustering analysis performed during the adsorption dynamics for the GolP-CHARMM case.

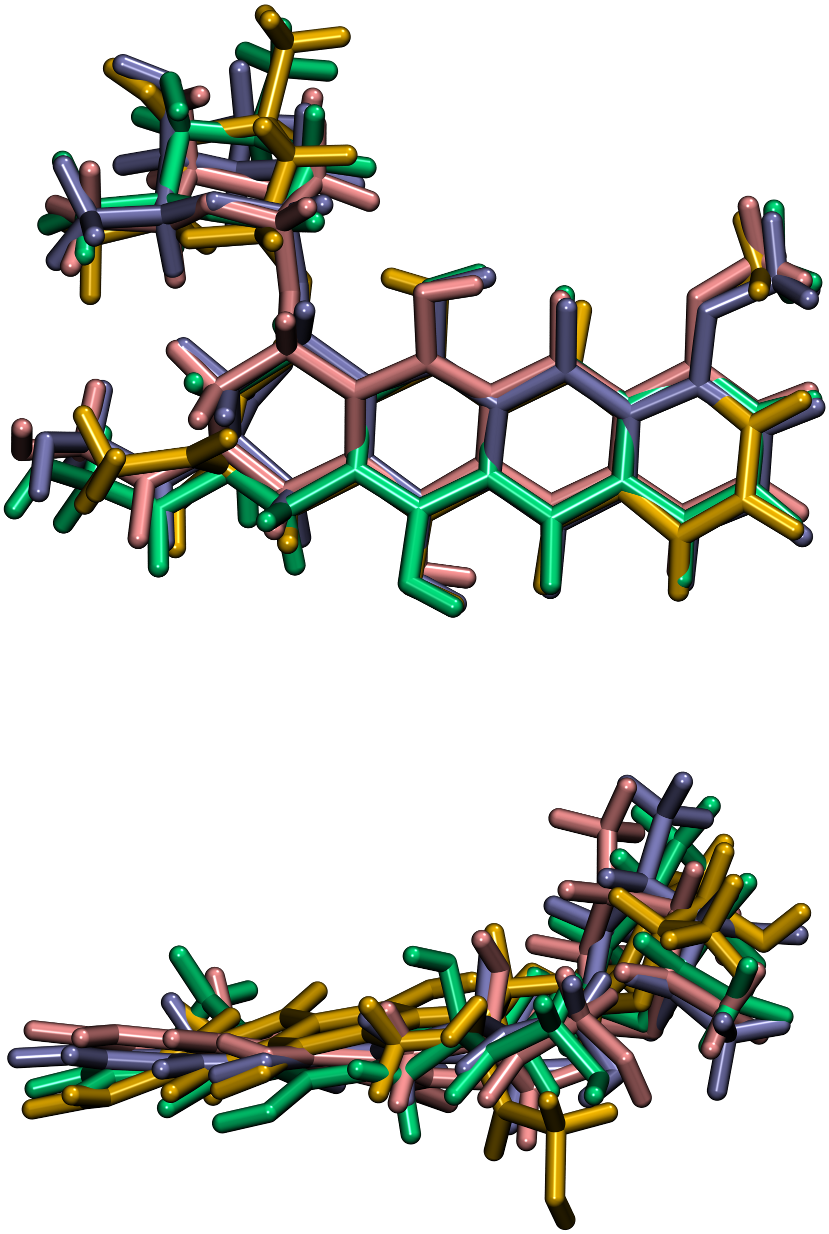

Figure S9: Superimposed adsorbed structures for each combination of FFs shown as top view (top image) and side view (bottom image). Color code: goldenrod for IFF/OPLS, pink for GolP, sea green for IFF/CHARMM and iceblue for GolP-CHARMM.

5 NCI analyses

Table S2: Integral values (in a.u.) for the Non-Covalent Interaction [8] surfaces of GUA. Values correspond to the integration over the volumes of the electron density, $\rho(\mathbf{r}) - \sum_i \rho_i(\mathbf{r})$ where i stands for the fragments: water, GUA, and gold surface.

Configuration	IFF/CHARMM	GolP-CHARMM
Free	1.09×10^{-3}	1.65×10^{-3}
Local minimum	2.07×10^{-3}	1.69×10^{-3}
TS	2.18×10^{-3}	3.00×10^{-3}
Global minimum	4.18×10^{-3}	2.14×10^{-3}

Table S3: Integral values (in a.u.) for the Non-Covalent Interaction surfaces of water molecules on Au(111) surfaces. Values correspond to the integration over the volumes of the electron density, $\rho(\mathbf{r}) - \sum_i \rho_i(\mathbf{r})$ where i stands for the fragments: water, and gold surface. See Figures S11 and S12 for visualizing the corresponding NCI surfaces.

	SPC	TIP3P
IFF(/OPLS)	2.78×10^{-2}	-
IFF(/CHARMM)	-	3.23×10^{-2}
GolP	2.18×10^{-2}	-
GolP-CHARMM	-	2.83×10^{-2}

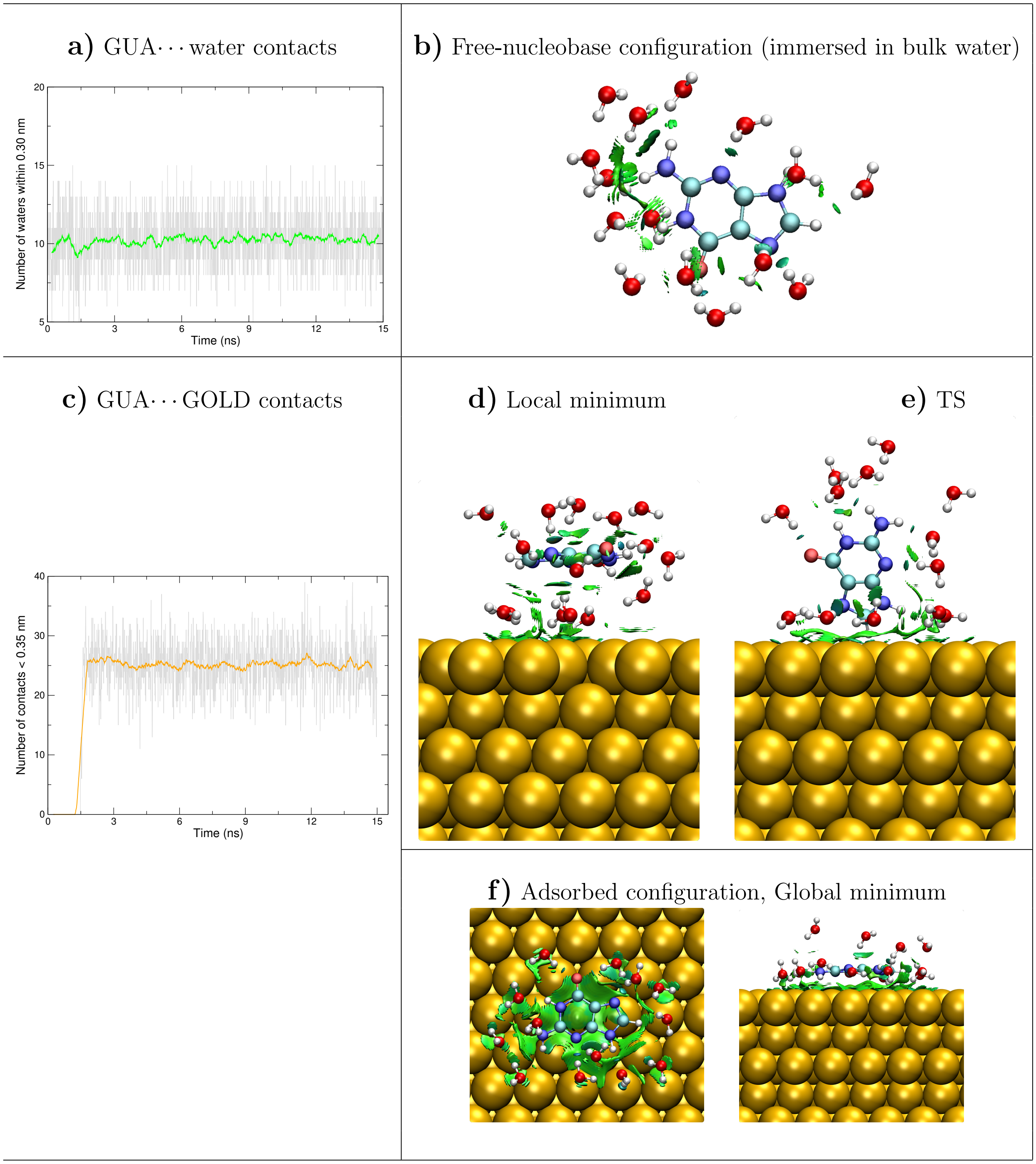

Figure S10: Analysis of the interactions occurring during the adsorption of guanine on the gold surface (IFF/CHARMM FF). Left: evolution in time of the number of contacts with the surface and water molecules surrounding guanine. Right: NCI plots of guanine in its in-solution state, local minimum, transition state, and global minimum (both top and lateral view) configurations. Water molecules within 0.3 nm of guanine are shown.

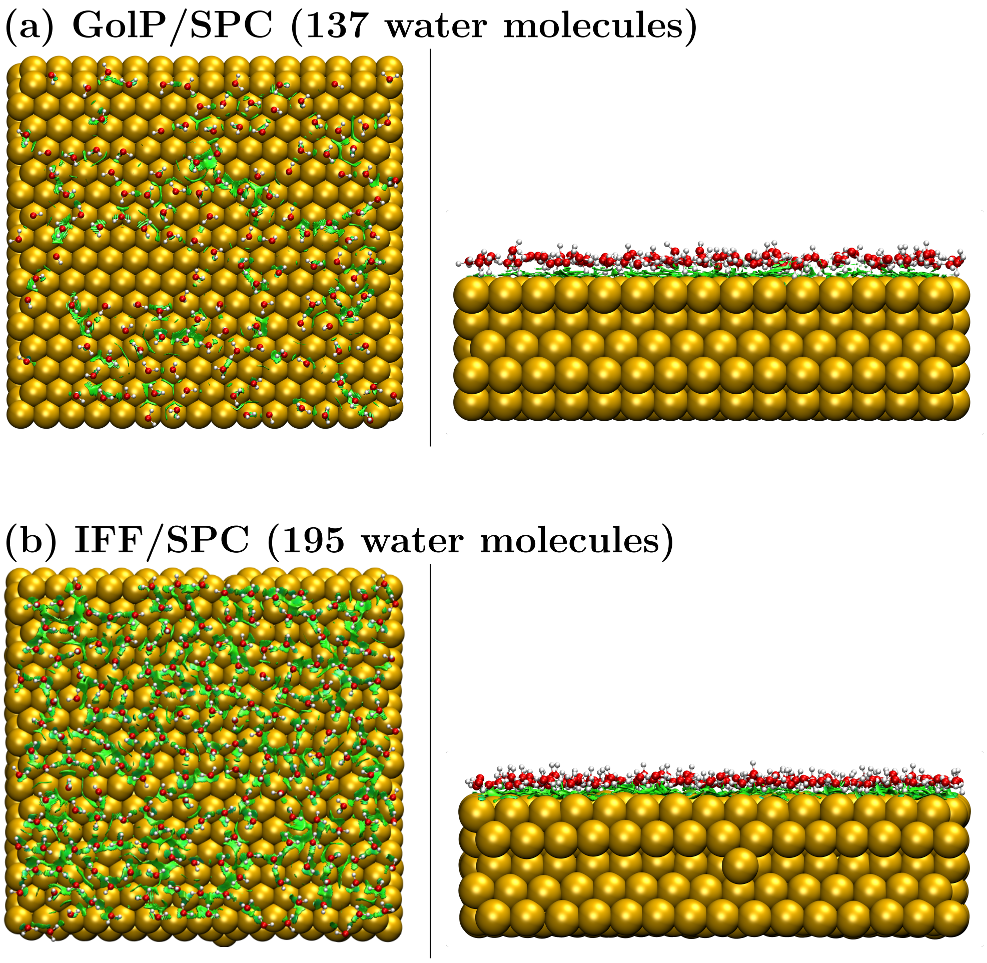

Figure S11: NCI plots of gold/water interfaces, in the absence of any adsorbate, in the (a) GolP/SPC and (b) IFF/SPC combinations. Water molecules within 0.35 nm from the gold slab are represented. Top views are shown on the left column and lateral views on the right column.

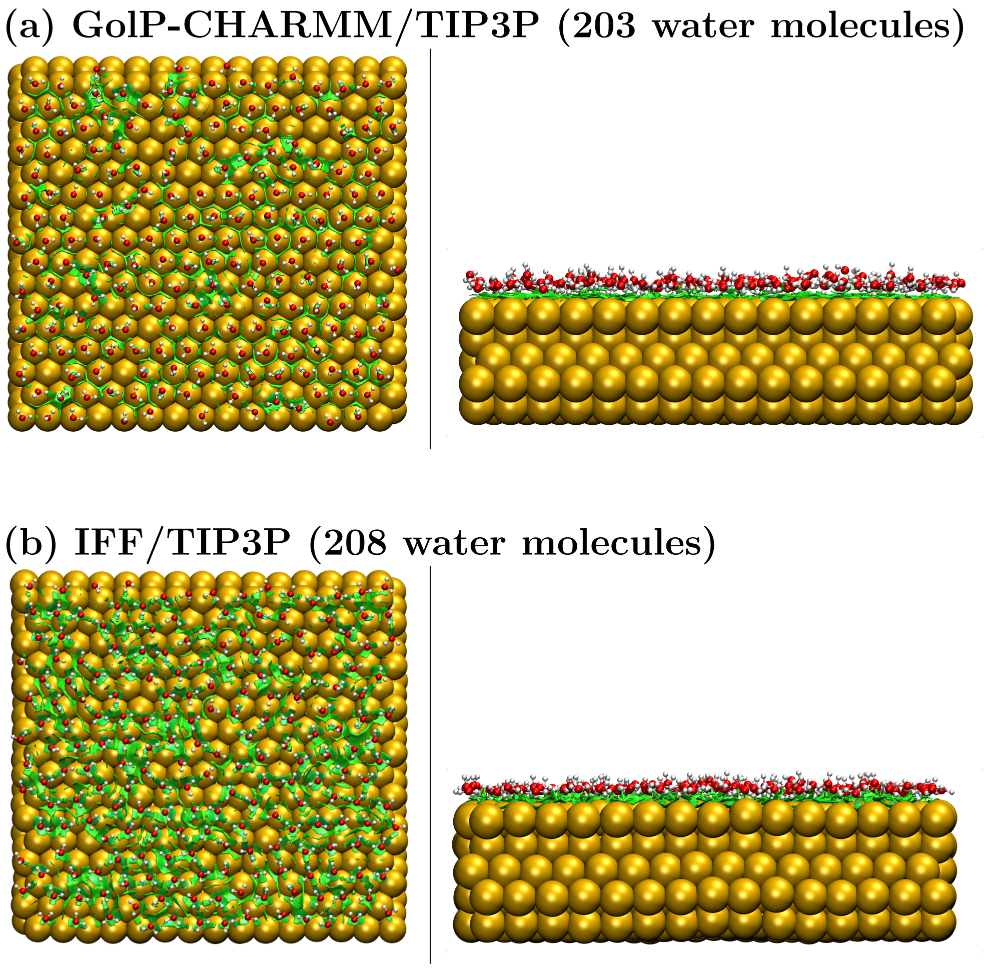

Figure S12: NCI plots of gold/water interfaces, in the absence of any adsorbate, in the (a) GolP-CHARMM/TIP3P and (b) IFF/TIP3P combinations. Water molecules within 0.35 nm from the gold slab are represented. Top views are shown on the left column and lateral views on the right column.

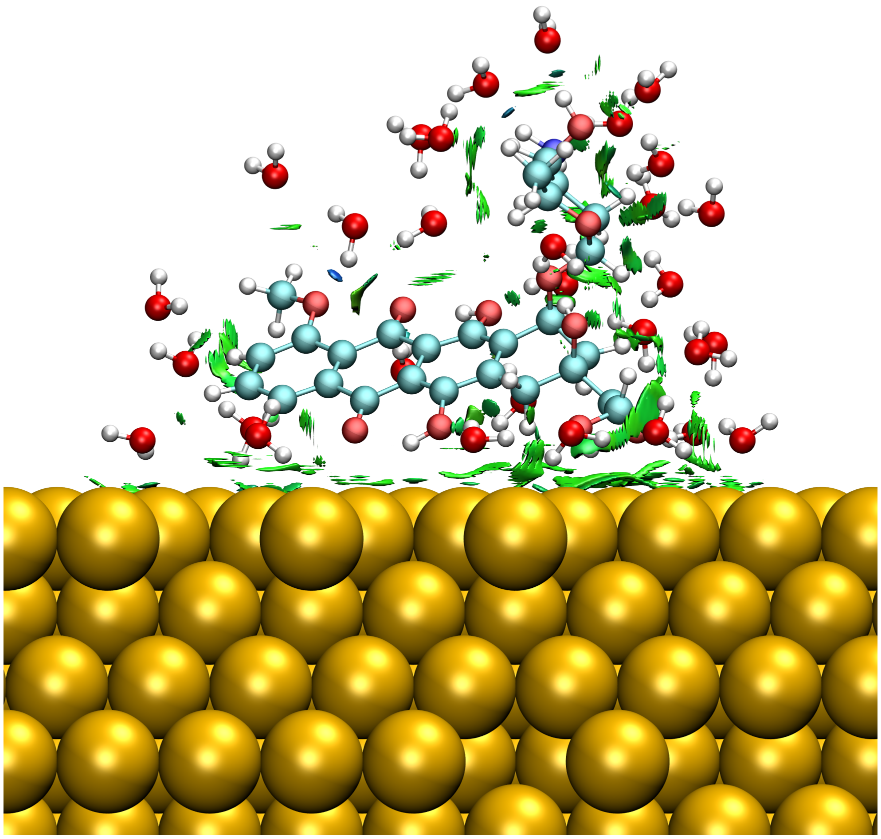

Figure S13: NCI plot of the transient configuration assumed by doxorubicin while approaching the gold surface in the GolP-CHARMM case.

References

[1] M. Hoefling, F. Iori, S. Corni, and K.-E. Gottschalk, “Interaction of amino acids with the au (111) surface: adsorption free energies from molecular dynamics simulations,” *Langmuir*, vol. 26, no. 11, pp. 8347–8351, 2010.

[2] L. Bellucci and S. Corni, “Interaction with a gold surface reshapes the free energy landscape of alanine dipeptide,” *J. Phys. Chem. C*, vol. 118, no. 21, pp. 11357–11364, 2014.

[3] Q. Shao and C. K. Hall, “Binding preferences of amino acids for gold nanoparticles: a molecular simulation study,” *Langmuir*, vol. 32, no. 31, pp. 7888–7896, 2016.

[4] Z. E. Hughes, L. B. Wright, and T. R. Walsh, “Biomolecular adsorption at aqueous silver interfaces: first-principles calculations, polarizable force-field simulations, and comparisons with gold,” *Langmuir*, vol. 29, no. 43, pp. 13217–13229, 2013.

[5] M. Rosa, R. Di Felice, and S. Corni, “Adsorption mechanisms of nucleobases on the hydrated au (111) surface,” *Langmuir*, vol. 34, no. 49, pp. 14749–14756, 2018.

[6] S. Rapino and F. Zerbetto, “Modeling the stability and the motion of dna nucleobases on the gold surface,” *Langmuir*, vol. 21, no. 6, pp. 2512–2518, 2005.

[7] Z. E. Hughes, G. Wei, K. L. Drew, L. Colombi Ciacchi, and T. R. Walsh, “Adsorption of dna fragments at aqueous graphite and au (111) via integration of experiment and simulation,” *Langmuir*, vol. 33, no. 39, pp. 10193–10204, 2017.

[8] R. A. Boto, F. Peccati, R. Laplaza, C. Quan, A. Carbone, J.-P. Piquemal, Y. Maday, and J. Contreras-García, “Nciplot4: Fast, robust, and quantitative analysis of noncovalent interactions,” *J. Chem. Theory Comput.*, vol. 16, no. 7, pp. 4150–4158, 2020.
